# Glucosamine links hyperglycemia to mTORC1 activation and glucose toxicity in diabetes

**DOI:** 10.1172/jci.insight.197331

**Published:** 2026-05-22

**Authors:** Yael Riahi, Aviram Kogot-Levin, Ziv Teselpapa, Elisheva Zemelman, Fatema Gamal, Tamar Cohen, Abed Nasereddin, Idit Shiff, Ifat Abramovich, Bella Agranovich, Dana Avrahami, Liad Hinden, Erol Cerasi, Daljeet Kaur, Lihi Grinberg, Ron Piran, Joseph Tam, Ernesto Bernal-Mizrachi, Erez Dror, Gil Leibowitz

**Affiliations:** 1 Diabetes Unit and Endocrine Service, Hadassah Medical Center, The Hebrew University, Jerusalem, Israel.; 2 The Genomic Applications Laboratory, The Core Research Facility, Faculty of Medicine, The Hebrew University, Jerusalem, Israel.; 3 The Laboratory for Metabolism in Health and Disease, Ruth and Bruce Rappaport Faculty of Medicine, Technion-Israel Institute of Technology Haifa, Israel.; 4 Department of Developmental Biology and Cancer Research, The Institute for Medical Research Israel-Canada, The Hebrew University-Hadassah Medical School, Jerusalem, Israel.; 5 Obesity and Metabolism Laboratory, Institute for Drug Research, School of Pharmacy, Faculty of Medicine, The Hebrew University, Jerusalem, Israel.; 6 The Azrieli Faculty of Medicine Bar Ilan University, Safed, Israel.; 7 Department of Internal Medicine, Division of Endocrinology, Metabolism and Diabetes, Miller School of Medicine, University of Miami, Miami, Florida, USA.

**Keywords:** Cell biology, Endocrinology, Metabolism, Beta cells, Diabetes, Signal transduction

## Abstract

Hyperglycemia is a principal driver of β cell failure and multiple-organ complications in diabetes. Chronic exposure to hyperglycemia overstimulates mTORC1, disrupting glucose metabolism and promoting ER stress, oxidative stress, and inflammation; however, the upstream metabolic signal(s) linking glucose to mTORC1 activation remains unclear. Here, we identified glucosamine as a key metabolite connecting elevated glucose to mTORC1 signaling in pancreatic islets and kidney, both major targets of hyperglycemic damage. Using ^13^C_6_-glucose metabolic labeling in diabetic rodents treated with or without the SGLT2 inhibitor dapagliflozin or insulin, combined with targeted metabolomics and metabolic flux analysis, we found that tissue glucose concentrations strongly correlated with glucosamine. A similar correlation with plasma glucose was conserved in humans with or without type 2 diabetes, and inversely associated with β cell function. In vitro, low-dose glucosamine stimulated mTORC1 in islets and kidney proximal tubule cells in an O-GlcNAcylation–dependent manner. Broad phosphoproteomics and transcriptomics analyses in β cells showed that glucosamine activated mTORC1-regulating pathways, induced oxidative stress, ER stress, and dedifferentiation. Genetic inhibition of β cell mTORC1 via heterozygous *Raptor* knockout, as well as pharmacologic inhibition of the glucosamine/mTORC1 axis through SGLT2 inhibition, alleviated β cell stress, improved glycemic control, and restored β cell function. These findings identified the glucosamine/mTORC1 pathway as an important mediator of β cell and kidney dysfunction in diabetes.

## Abstract 2

In diabetes glucosamine is de novo generated from glucose and accumulates in multiple tissues, including in beta-cells. Glucosamine stimulates mTORC1 and induces beta-cell dysfunction. Inhibiting the glucosamine-mTORC1 pathway improves diabetes and beta-cell function.

## Graphical abstract

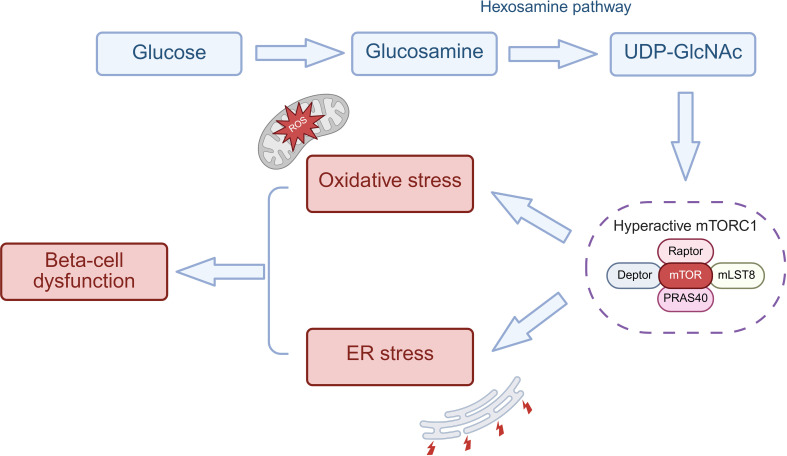

## Introduction

Diabetes is a major health problem resulting from the progressive decline in β cell function, leading to hyperglycemia and subsequent end-organ complications (1). Diabetic kidney disease (DKD) is a common complication of diabetes, occurring in 20%–40% of patients (2). It is the leading cause of end-stage renal disease in the Western world and markedly increases mortality risk (3). Clinical trials have consistently shown that reducing hyperglycemia, i.e., glucotoxicity, improves β cell function and prevents diabetes complications, including DKD (4–6).

Hyperglycemia may promote ER stress, oxidative stress, and inflammation observed in islets as well as in other tissues through sustained activation of the key nutrient sensor mTORC1 (7, 8). Identifying the mechanisms underlying glucose regulation of mTORC1 is challenging because glucose is metabolized via glycolysis and the tricarboxylic acid (TCA) cycle, affecting ATP generation, redox state, and the levels of various glycolytic intermediates and amino acids (AAs). The ATP/AMP ratio can modulate mTORC1 activity through AMPK, whereas AAs, and likely carbohydrates, signal to mTORC1 via the Rag family of GTPases (9–11). Glucose metabolism varies across tissues and cell types, complicating the question of how glucose regulates mTORC1. We and others have previously shown that mTORC1 activity in islet cells and in kidney proximal tubule cells (KPTCs) plays a central role in mediating β cell, α cell, and kidney dysfunction in diabetes (7, 8, 12–16). Pancreatic β cells and KPTCs represent 2 complementary regulators of whole-body glucose homeostasis; while β cells control glycemia through insulin secretion, KPTCs regulate glucose clearance through reabsorption. Both cell types are highly sensitive to nutrient availability and metabolic stress, making them ideal models for investigating mTOR signaling and nutrient sensing pathways.

In β cells, glucose transport is mediated by GLUT2 (GLUT1/3 in humans) and followed by phosphorylation by glucokinase, which has a low affinity for glucose and is not inhibited by its product, glucose-6-phosphate (G6P). These properties allow tight coupling between plasma glucose levels, glycolysis, and glucose oxidation for energy production, thereby regulating insulin secretion (17). In contrast, in KPTCs, glucose transport occurs through sodium-glucose cotransporters (SGLTs, primarily SGLT2) on the luminal side and various glucose transporters on the basolateral side. Glucose is phosphorylated by hexokinase, which has high affinity for glucose and is inhibited by G6P. Glucose is poorly oxidized in the TCA cycle, and most of the glucose exits the cells via GLUT2 (18, 19). Studying the mechanisms of mTORC1 activation by hyperglycemia in cells with distinct glucose utilization and oxidation patterns may help to identify novel regulators of mTORC1 that act independently of glycolysis and glucose oxidation in the TCA cycle. Therefore, we investigated how glucose regulates mTORC1 in these cell types with the goal of identifying a common mechanism underlying mTORC1 activation during chronic hyperglycemia.

Here, we performed in vivo metabolic labeling using ^13^C_6_-glucose in normoglycemic and diabetic animals treated with or without glucose-lowering agents, followed by broad, unbiased targeted metabolomics across multiple organs. We analyzed correlations between tissue glucose levels and various mTORC1-regulating metabolites and tested their effects on diabetes and β cell function. Our results identified glucosamine as a key regulatory metabolite. This amino sugar, which serves as the biochemical precursor to all nitrogen-containing sugars (20), is essential for the synthesis of glycosylated proteins and lipids.

We found that glucosamine is a ubiquitous sensor of tissue glucose levels and partially mediates glucose toxicity in diabetes through sustained stimulation of mTORC1 and induction of ER stress and oxidative stress. Genetic inhibition of mTORC1 in adult β cells ameliorated ER stress–induced diabetes. We propose that SGLT2 inhibitors (SGLT2is) may improve diabetes outcomes and β cell function by suppressing the glucosamine/mTORC1 pathway.

## Results

### Glucosamine synthesis and metabolism in diabetes.

We treated WT mice and diabetic Akita mice with dapagliflozin or insulin for 5–7 days followed by metabolic labeling with ^13^C_6_-glucose, and performed metabolomics and metabolic flux analysis in kidney cortex extracts, liver, heart, and plasma. The full list of metabolites in the different tissues of WT and diabetic animals and their response to treatment has been published and is available online (16).

**Figure 1:**
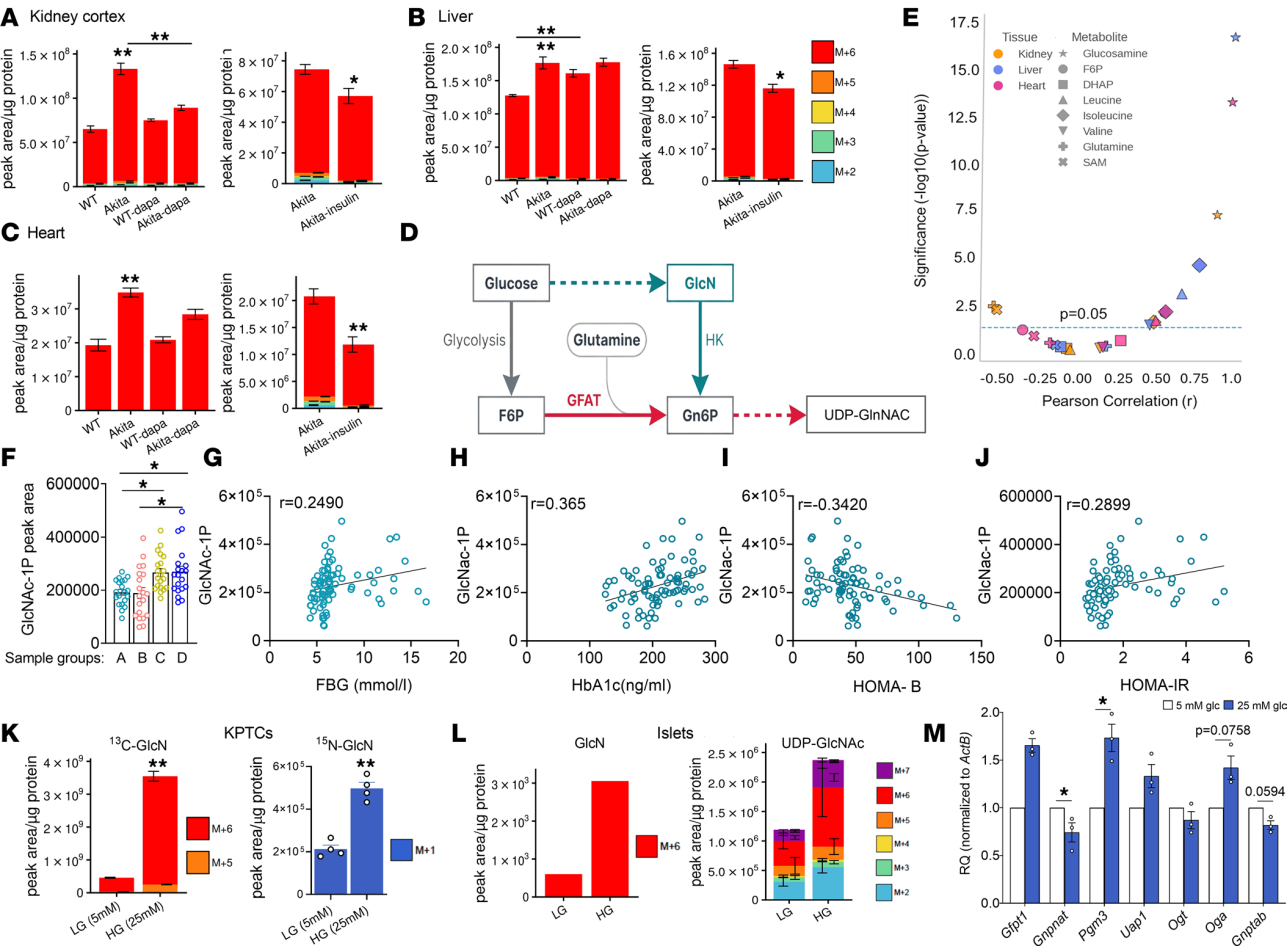
Glucosamine synthesis and metabolism in diabetes. WT and Akita mice were treated with and without dapagliflozin or insulin for 1 week, followed by ^13^C-glucose injections and metabolomics analysis. (**A**–**C**) Relative abundance of ^13^C-labeled glucosamine levels in kidney cortex, liver, and heart. Data represent the mean ± SEM, *n* = 4–6 mice per group. A Student’s *t* test was used for 2-group comparisons, and 1-way ANOVA for multiple-group comparisons. (**D**) Schematic representation of Gn6P and UDP-GlcNAc synthesis through the canonical and non-canonical pathways. (**E**) Correlation between glucose and glucose-derived metabolites (glucosamine, F6P, DHAP, leucine, isoleucine, valine, glutamine, and *S*-adenosylmethionine [SAM] levels) in the kidney cortex, liver, and heart. Pearson’s correlation coefficient (*r*) was determined via linear regression. (**F**–**J**) Hexosamine pathway metabolites in plasma of human patients with different FBG categories (**A**: <5.6 mmol/L, **B**: 5.6–6.1 mmol/L, **C**: 6.1–7 mM (IFG), **D**: ≥7.0 mM [diabetes]). (**F**) Relative GlcNAc-1p levels; *n* = 20 per group. Data were analyzed by 1-way ANOVA. (**G**–**J**) Correlation between GlcNAc-1P and fasting blood glucose HbA1c, HOMA-B, and HOMA-IR. Pearson’s correlation coefficient (*r*) was determined via linear regression. (**K**–**L**) KPTCs and human islets were cultured at low glucose (LG, 5 mM) or high glucose (HG, 25 mM) for 48 hours, followed by labeling with ^13^C-glucose for 3 hours or with 4 mM L-glutamine-^13^C_5_,^15^N_2_ for 6 hours. (**K**) ^13^C-glucosamine and ^15^N-glucosamine levels in primary KPTCs (*n* = 3 per group). Data were analyzed by Student’s *t* test. (**L**) ^13^C-glucosamine and UDP-GlcNAc levels in islets. (**M**) RT-qPCR of enzymes involved in the hexosamine pathway in mouse islets incubated at 5 mM or 25 mM glucose for 72 hours (*n* = 3 per group). Data were analyzed by 1-tailed Student’s *t* test. *P < 0.05; **P < 0.01.

Glucosamine was one of the top metabolites elevated in diabetic animals, showing 1.5- to 2-fold higher levels in kidney, liver, and heart extracts; treatment with dapagliflozin or insulin decreased the glucosamine levels (Figure 1, A–C), suggesting that this elevation is a consequence of hyperglycemia.

In humans, glucosamine is present in the form of glucosamine-6-phosphate (Gn6P). The current view is that Gn6P is synthesized from fructose-6-phosphate (F6P) and glutamine by glutamine-fructose-6-phosphate transaminase (GFAT) as the first step of the hexosamine pathway (Figure 1D) (21). The end-product of this pathway is uridine diphosphate–N-acetylglucosamine (UDP-GlcNAc), which is used for generating glycosaminoglycans, proteoglycans, and glycolipids, and for posttranslational modification by glycosylation (22).

Surprisingly, ^13^C_6_-glucose labeling showed that all 6 glucosamine carbons were labeled with the ^13^C-isotopologue, indicating that glucosamine is directly synthesized from glucose; the fraction of M+6 constituted 94.3%–100% of all labeled glucosamine (Figure 1, A–C). A summary of the full mass isotopologue distributions from all experiments is provided in Supplemental Table 1. Glucosamine levels in different tissues strongly correlated with the glucose concentration (*r* = 0.86–0.98) (Figure 1E). Of note, the correlation between tissue glucose and glycolytic intermediates, including F6P and dihydroxyacetone-phosphate (DHAP), branched-chain AAs and additional AAs that regulate mTORC1, was weaker than that of glucosamine and varied between tissues (Figure 1E). To explore the relevance of altered glucosamine metabolism to type 2 diabetes (T2D) in humans, we analyzed previously reported plasma metabolomics data of patients with impaired glucose tolerance (IGT) and/or impaired fasting glucose (IFG) and T2D (23). The glucosamine metabolite GlcNAc-1P was increased in the plasma of patients with IFG and T2D (Figure 1F). GlcNAc-1P correlated with fasting glucose, HbA1c, and homeostatic model assessment for insulin resistance (HOMA-IR) (Figure 1, G, H, and J), and was inversely correlated with HOMA-B, a marker of β cell function (Figure 1I). Collectively, these data suggest that glucosamine/GlcNAc is associated with both β cell dysfunction and insulin resistance.

We further studied glucosamine synthesis and metabolism in primary KPTCs (Figure 1K) and human islets (Figure 1L and Supplemental Figure 1; supplemental material available online with this article; https://doi.org/10.1172/jci.insight.197331DS1) cultured under chronic low glucose (LG) or high glucose (HG). Consistent with the in vivo data, exposure of both islets and KPTCs to HG markedly increased glucosamine levels and approximately 100% of the glucosamine molecules were labeled with the ^13^C-isotopologue at all 6 carbons (M+6), indicating endogenous synthesis from glucose. Exposure of KPTCs to HG increased ^13^C_6_-glucosamine levels by approximately 3-fold; metabolic labeling with L-glutamine-^13^C_5_,^15^N_2_ demonstrated that the amine group is derived from glutamine (Figure 1K). Similarly, in human islets, exposure to HG increased ^13^C_6_-labeled and total glucosamine by 6-fold and UDP-GlcNAc levels by 2-fold (Figure 1L and Supplemental Figure 1). Notably, chronic incubation of mouse islets under HG increased the expression of different hexosamine pathway enzymes, including *Gfat1*, *Pgm3*, and *Oga*, suggesting that chronic exposure to HG also modulates the activity of this pathway (Figure 1M).

**Figure 2:**
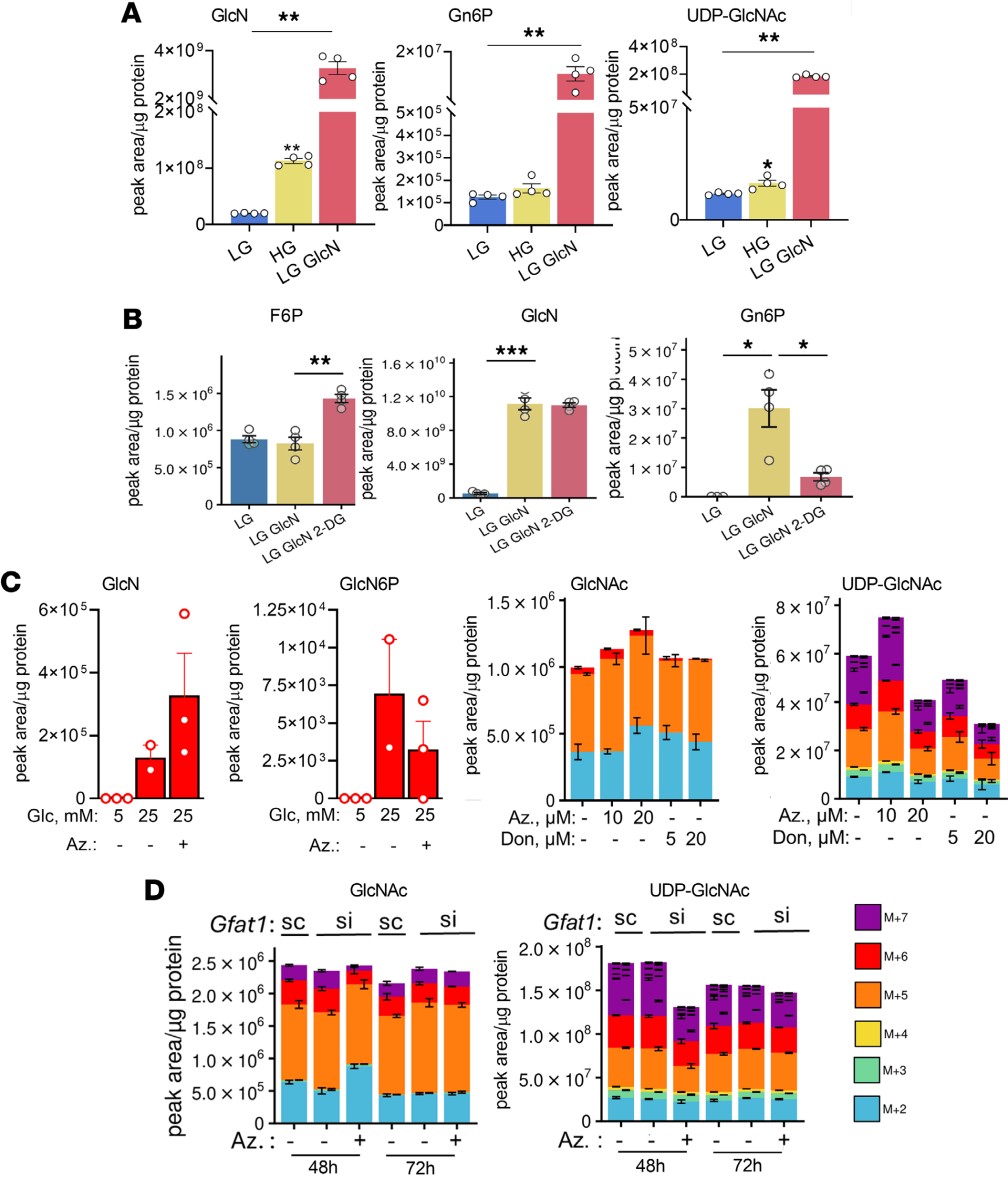
Hexosamine pathway metabolites. (**A**) Levels of glucosamine (GlcN) and hexosamine pathway metabolites in primary KPTCs cultured at LG, HG, or LG with GlcN (5 mM) for 48 hours (*n* = 4 per group). Data were analyzed by 1-way ANOVA. (**B**) Levels of F6P, GlcN, and Gn6P in primary KPTCs cultured at LG, LG with GlcN (5 mM), or GlcN and 2-DG (5 mM) for 6 hours (*n* = 3 per group). Data were analyzed by 1-way ANOVA. *P < 0.05; **P < 0.01; ***P < 0.001. (**C**) MIN6 cells were incubated at LG or at HG with or without the GFAT inhibitor azaserine (Az.) or 6-diazo-5-oxo-L-norleucine (DON) at the indicated concentrations followed by metabolic labeling with a similar concentration of ^13^C_6_-glucose for 3 hours. ^13^C-labeled hexosamine pathway metabolites are shown (*n* = 3 per group). (**D**) MIN6 cells were transfected with *Gfat1* siRNA oligonucleotides (si) or with scrambled oligonucleotides (sc; control) and then treated with or without azaserine (5 mM). Forty-eight or 72 hours after transfection, metabolic labeling was performed as in **C** (*n* = 3 per group). ^13^C-labeled hexosamine pathway metabolites are shown.

We next investigated glucosamine metabolism in KPTCs and human islets treated with glucosamine followed by liquid chromatography–mass spectrometry (LCMS). Glucosamine markedly increased levels of Gn6P and UDP-GlcNAc in both KPTCs and human islets, indicating that glucosamine enters the hexosamine pathway via phosphorylation to Gn6P with subsequent generation of the end-product UDP-GlcNAc (Figure 2A and Supplemental Figure 1A). Importantly the area under the curve (AUC) of glucosamine levels in human islets incubated with 0.3 mM glucosamine was like that of human islets incubated with HG (25 mM; Supplemental Figure 1A). In extracts of human islets cultured under LG conditions, the intracellular glucose concentration was 25.4 nM/mg of protein. The corresponding glucosamine concentration was 0.98 nM/mg of protein, representing approximately 4% of the intracellular glucose level (Supplemental Figure 1B). This proportion is consistent with the established fraction of glucose metabolized through the hexosamine pathway (24), indicating that the exogenous glucosamine levels used in this study effectively mimic the intracellular environment seen in HG conditions in vitro and in vivo.

It has been previously suggested that glucosamine is phosphorylated to Gn6P by hexokinases (25). Consistently, we found that treatment of KPTCs with the hexokinase inhibitor 2-deoxyglucose (2-DG) effectively inhibited glucosamine phosphorylation to Gn6P, although F6P levels increased (Figure 2B). 2-DG inhibits G6P isomerase, which may explain the accumulation of F6P despite hexokinase inhibition. In any case, the marked reduction of Gn6P by 2-DG under conditions where F6P is increased supports the hypothesis that Gn6P can be generated by direct phosphorylation of glucosamine, rather than via GFAT. To rigorously test this hypothesis and distinguish between the canonical GFAT-dependent pathway and this non-canonical route, we inhibited GFAT in MIN6 cells cultured under HG conditions using both pharmacological inhibitors and genetic knockdown. Treatment with the GFAT inhibitor azaserine or 6-diazo-5-oxo-L-norleucine (DON) did not alter the levels of ^13^C_6_-glucose–derived hexosamine metabolites (GlcN, Gn6P, GlcNAc, and UDP-GlcNAc) (Figure 2C). Additionally, siRNA-mediated knockdown of *Gfat1* reduced its expression by approximately 40%; to compensate for the partial knockdown, we inhibited residual GFAT activity by cotreatment with azaserine. Neither *Gfat1* knockdown alone nor in combination with azaserine affected GlcNAc or UDP-GlcNAc levels (Figure 2D). Taken together, these results provide strong evidence for a non-canonical, GFAT-independent hexosamine synthesis pathway.

Collectively, we show the presence of a non-canonical hexosamine pathway, in which glucosamine is generated de novo from glucose and glutamine followed by its phosphorylation to Gn6P to feed the hexosamine pathway (Figure 1D).

### Phosphoproteomic analysis of the glucosamine-OGT effects on signal transduction focusing on mTORC1.

O-GlcNAcylation is a posttranslational modification, responsive to nutrient availability and stress, involving the attachment of O-linked GlcNAc moieties to serine and threonine residues of cytoplasmic and mitochondrial proteins (26, 27). This process is regulated by a single pair of enzymes — O-GlcNAcyl transferase (OGT) and O-GlcNAcase (OGA) — that tightly control the dynamic cycling of this modification (28). Balanced O-GlcNAcylation is required for β cell and kidney function (29–34). Consistently, both hypo-O-GlcNAcylation by *Ogt* knockout (KO) or hyper-GlcNAcylation by *Oga* KO are deleterious (29–34). Mechanistically, OGT utilizes UDP-GlcNAc as a substrate, which is generated via the hexosamine biosynthetic pathway (HBP). Exogenous glucosamine enters the HBP downstream of the rate-limiting enzyme GFAT, rapidly elevating UDP-GlcNAc levels and driving global O-GlcNAcylation.

**Figure 3:**
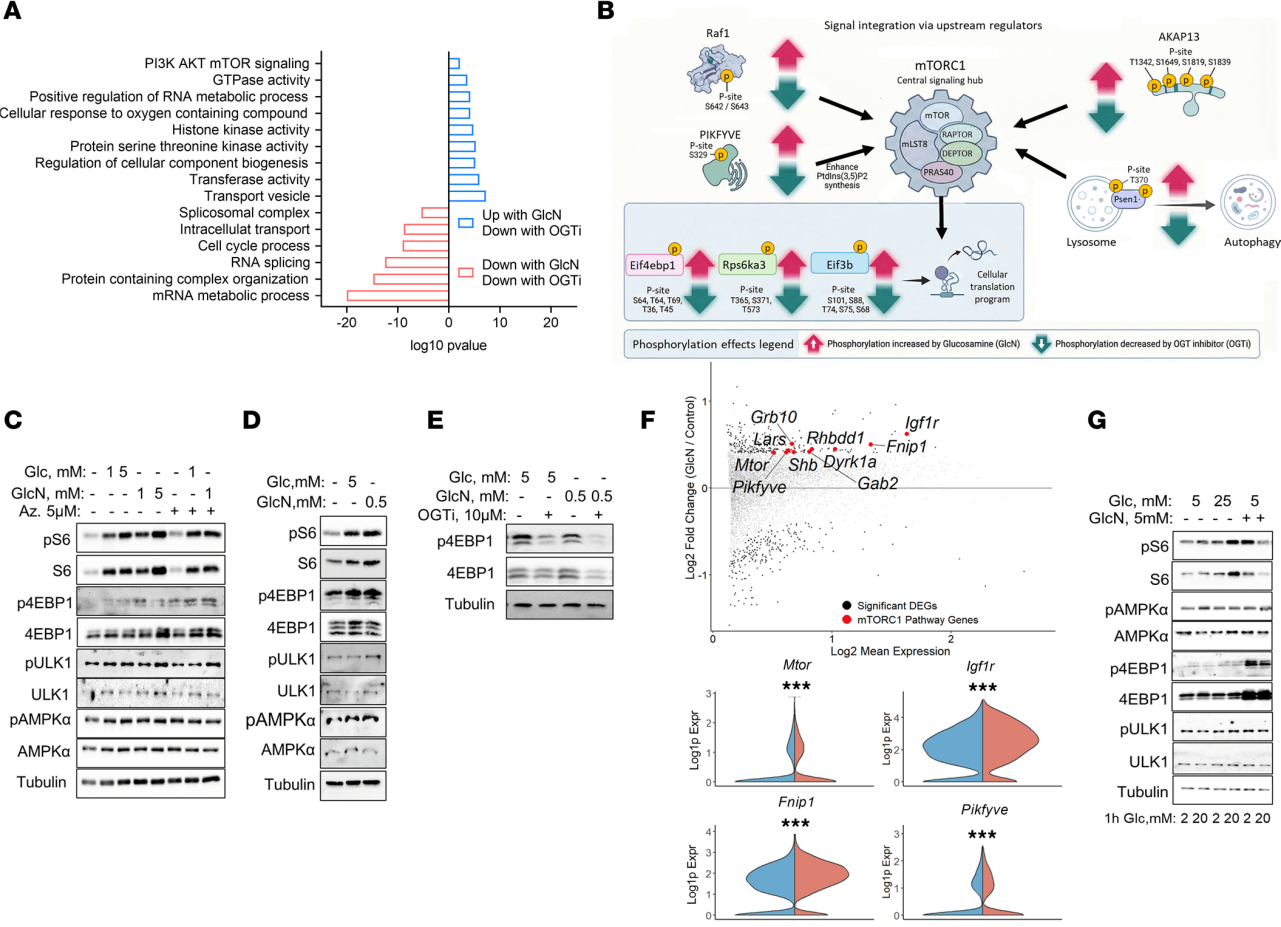
Regulation of β cell mTORC1 by glucosamine. Phosphoproteomics in MIN6 cells treated with glucosamine (GlcN) with or without the OGT inhibitor (OGTi) ST 045849. MIN6 cells were serum starved for 2 hours and stimulated for 30 minutes with GlcN (1 mM) ± OGTi (10 μM). In the OGTi group, cells were pretreated with OGTi for 2 hours. (**A**) Pathway enrichment analysis is shown, with bars representing processes that have significant phosphorylation changes (*n* = 3 per group). Data were analyzed by Student’s *t* test. Blue bars denote pathways where phosphorylation is increased by GlcN and reverted by OGTi; red bars denote the opposite pattern. (**B**) Schematic model of the mTORC1 signaling hub summarizing key GlcN-induced phosphorylation events (red arrows) and their reversal by OGTi (green arrows). (**C** and **D**) Western blot analysis of the mTORC1 and AMPK activity in MIN6 cells (**C**) and mouse islets (**D**). Following a 2-hour starvation, cells and islets were stimulated for 30 minutes with glucose (Glc) or GlcN. One group included azaserine throughout. Blots show phosphorylated and total S6, 4EBP1, ULK1, and AMPK (**C**, *n* = 3–7 per group; **D**, *n* = 2, 200 islets per treatment were pooled from 3 mice). (**E**) mTORC1 activity in MIN6 cells, incubated in starvation medium for 2 hours, followed by treatment with Glc (5 mM) or GlcN (0.5 mM), ± OGTi (10 mM) (*n* = 3 per group). (**F**) snRNA-seq on cells from islets chronically treated with GlcN. MA plot and violin plots showing GlcN’s effects on the expression of mTOR-regulating genes (*n* = 3 per group). DEGs, differentially expressed genes. Data were analyzed by the Wilcoxon rank-sum test. ***P < 0.001. (**G**) MIN6 cells were incubated at LG with or without GlcN or HG for 72 hours followed by a 1-hour incubation at 2 or 20 mM glucose. Western blotting for phosphorylated and total S6, 4EBP1, ULK1, and AMPK was conducted (*n* = 3 per group).

To study the downstream effect of this metabolic flux in β cells, we sought to investigate the changes in the immediate response to glucosamine and how it is affected by inhibition of OGT. Thus, we performed a phosphoproteomic analysis in cells treated with low-dose glucosamine with or without an OGT inhibitor (OGTi; ST 045849, 10 μM). Interestingly, gene set enrichment analysis (GSEA) revealed that glucosamine treatment significantly enriched the mTORC1 signaling pathway, an effect that was prevented by OGTi (Figure 3A). Focused analysis of this pathway discovered that the phosphorylation of key regulatory nodes of mTORC1 is regulated by glucosamine in an OGT-dependent manner (Figure 3B). Glucosamine increased the phosphorylation of upstream regulators of mTORC1, including the MAPK/ERK protein kinase Raf1 (35, 36) and the PKA-scaffolding protein Akap13, which mediates PKA signaling to mTORC1 (37). Additionally, phosphorylation of the phosphoinositide-producing kinase PIKFYVE (38, 39) was activated by glucosamine through O-GlcNAcylation. PIKFYVE regulates mTORC1 primarily through enzymatic production of phosphatidylinositol 3,5-biphsphate, which facilitates mTORC1 translocation to lysosomes, where mTORC1 is activated in response to nutrients. Glucosamine increased the phosphorylation of presenilin-1 (Psen1) at T370, a conserved residue within its cytosolic loop (40). Psen1 mutations account for the most common cause of familial Alzheimer disease. Of note, *Psen1* knockin mutations have been shown to induce sustained activation of mTORC1 (31). Downstream effectors of mTORC1, including S6K1 and 4E-BP1, along with subunits of the EIF3 initiation factor (41), were hyperphosphorylated by glucosamine, whereas OGT inhibition abrogated it. Western blot analysis confirmed that glucosamine acutely stimulated mTORC1 activity in both MIN6 β cells and islets (Figure 3, C and D, and Supplemental Figure 2, A–C). Specifically, acute treatment with glucosamine or glucose increased the phosphorylation of S6 and 4EBP1 (without affecting ULK1 and AMPK phosphorylation; Figure 3, C and D). The OGTi inhibited mTORC1 (4EBP1 phosphorylation) in β cells treated with glucosamine (Figure 3E). Importantly, the GFAT inhibitor azaserine and the siRNA-mediated *Gfat1* knockdown did not affect the mTORC1 response to glucose and glucosamine (Figure 3C and Supplemental Figure 3A).

To evaluate potential changes in the transcriptome of β cells, we profiled dispersed islets at single-nucleus resolution (snRNA-seq). This approach enabled us to specifically analyze β cells following chronic glucosamine treatment, identifying transcriptional changes that may contribute to sustained activation of mTORC1. Of note, glucosamine increased the expression of mTOR, as well as genes involved in growth factor signaling, e.g., *Igf1r* (42), *Pikfye* (38), *Fnip1* (folliculin-interacting peptide) (43, 44), and *Lars1* (leucyl-tRNA synthetase 1, LARS1), which regulates nutrient sensing via the RAG protein complex (45) (Figure 3F). Western blot analysis confirmed that chronic treatment with glucosamine stimulated mTORC1, evident by increased S6 and 4EBP1 phosphorylation and protein levels (Figure 3G and Supplemental Figure 2C). Phosphorylation of 4E-BP1 causes its release from eIF4E to allow cap-dependent translation. We hypothesized that sustained phosphorylation of 4EBP1 requires an adaptive increase in 4EBP1 level to allow adequate regulation of protein synthesis under conditions of increased demand. We tested this hypothesis by incubating MIN6 cells with glucosamine with and without low-dose (10 nM) rapamycin for 48 hours. Treatment with rapamycin markedly decreased 4EBP1 levels, indicating that both 4EBP1 synthesis and phosphorylation are regulated by mTORC1 (Supplemental Figure 3B). Treatment with HG or glucosamine promoted mTORC1 localization to the lysosome, as evidenced by the increased colocalization of mTORC1 with LAMP2 (Supplemental Figure 3C), further suggesting mTORC1 activation.

**Figure 4:**
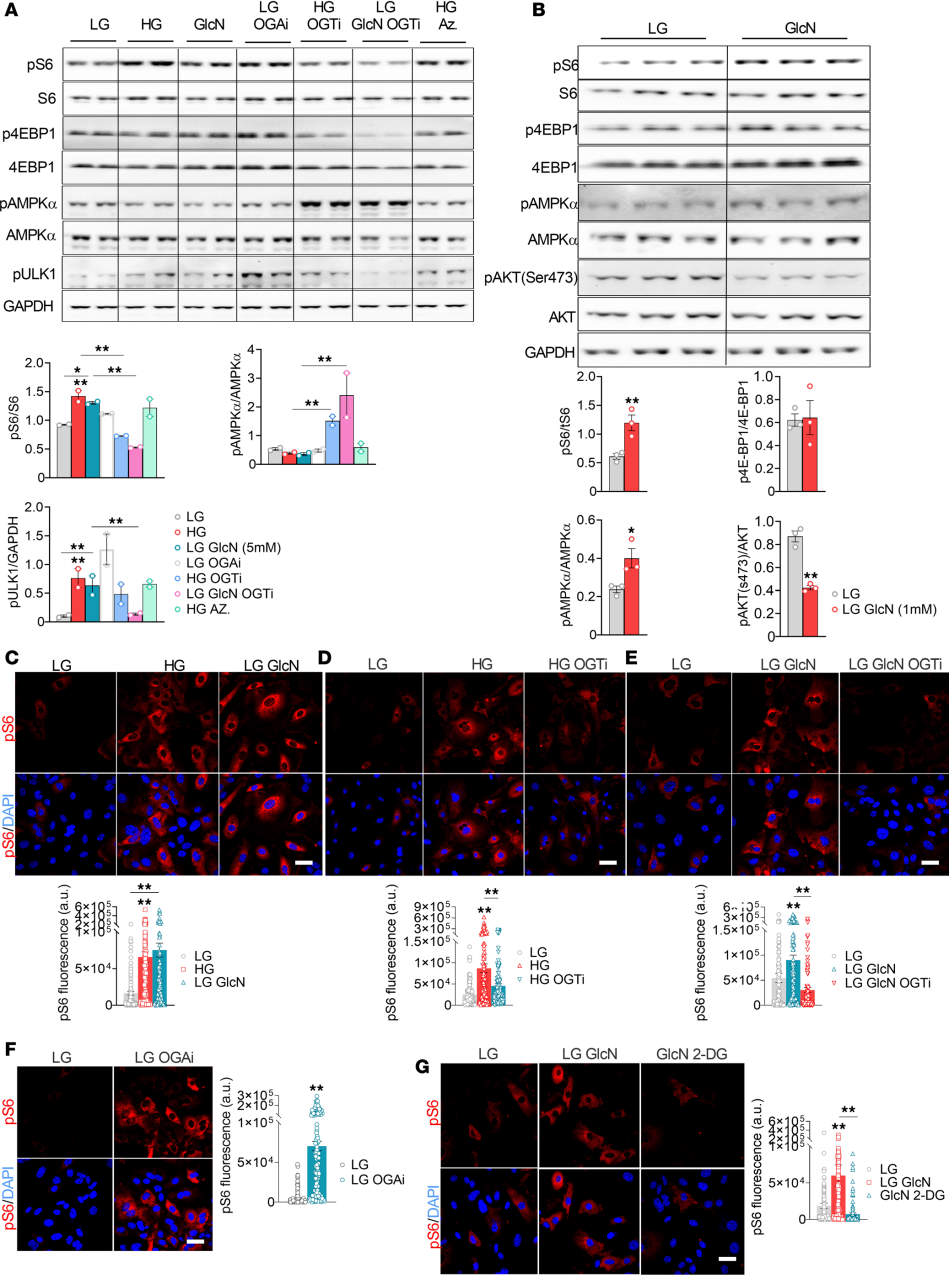
Regulation of mTORC1 by glucosamine in KPTCs. (**A** and **B**) mTORC1 activity in HK2 cells (**A**) and in primary KPTCs (**B**). Cells were starved for 2 hours, and then incubated at LG or HG, with or without glucosamine (GlcN), the OGA inhibitor (OGAi) TMG (15 μmol/L), OGTi (10 μM), or the GFAT inhibitor azaserine (5 μM) for 30 minutes (*n* = 2–3 per group). (**C**–**G**) Representative immunofluorescence images showing phosphorylated S6 (p-S6) in KPTCs after 48 hours of culture. KPTCs were incubated at LG or HG, with or without GlcN (5 mM), OGTi (10 μM), OGAi (15 μM), or 2-deoxyglucose (2-DG) (*n* = 3–6 per group). Images were acquired at ×60 magnification. *P < 0.05; **P < 0.01 by Student’s *t* test (**B** and **F**) or 1-way ANOVA (**A**, **C**, and **G**). Scale bars: 10 μm.

Similarly, Western blotting in HK2 cells (human KPTC line) and in primary KPTCs and immunostaining showed that both HG and glucosamine stimulated mTORC1 in an OGT-dependent manner, whereas azaserine did not affect the glucose stimulation of mTORC1 (Figure 4, A–E). The OGA inhibitor TMG increased mTORC1 activity under LG, indicating that induction of O-linked GlcNAcylation is sufficient to activate mTORC1 (Figure 4F). We found that inhibiting hexokinase with 2-DG prevented glucosamine metabolism in the hexosamine pathway (Figure 2B). Consistently, 2-DG abrogated the stimulation of mTORC1 by glucosamine (Figure 4G). Collectively, we show that glucosamine induces sustained activation of mTORC1 in an OGT-dependent manner. The glucosamine regulation of mTORC1 is probably mediated through multiple effects on protein posttranslational modifications and gene expression.

### The glucosamine/mTORC1 pathway regulates glucose metabolism and mitochondrial function in KPTCs.

Incubation of KPTCs in medium containing a high glucosamine concentration (5 mM) effectively stimulated mTORC1 (Figure 4, C, E, and G). Under these conditions, glycolysis and glucose oxidation downstream of phosphofructokinase (PFK) were inhibited, evident by decreased fructose 1,6-biphosphate (F1,6-BP), DHAP/glyceraldehyde-3-phosphate (G3P), phosphoenolpyruvate (PEP), and lactate, and of TCA cycle metabolites (Supplemental Figure 4, A and B), as was previously shown (46). Seahorse experiments further showed that lactate production (extracellular acidification rate) decreased, along with impaired basal and ATP-linked respiration (Supplemental Figure 4, C and D). These findings indicate that glucosamine stimulates mTORC1 even under conditions where glycolysis and glucose oxidation are inhibited.

**Figure 5:**
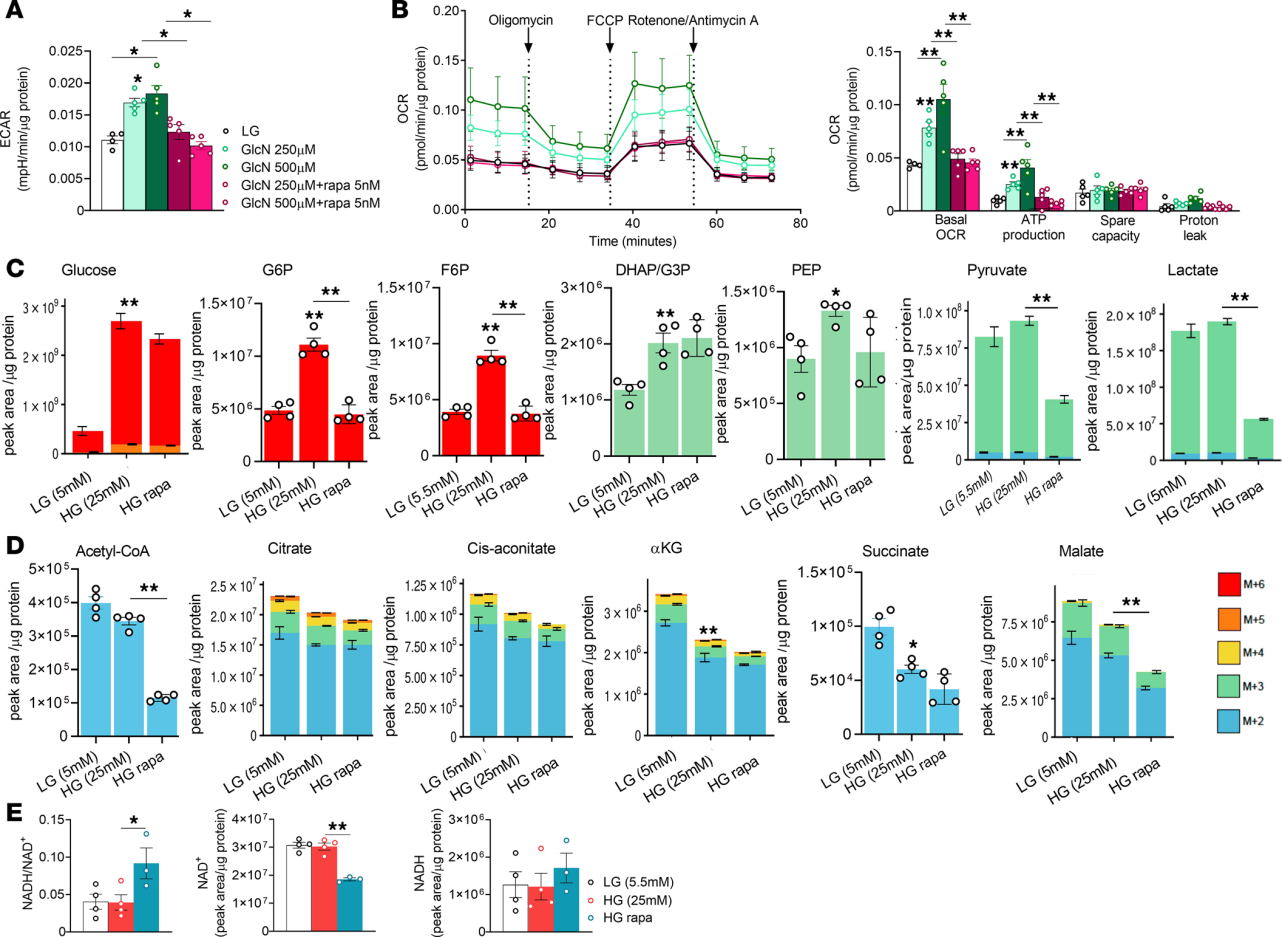
Regulation of glucose metabolism and mitochondrial function in KPTCs via the glucosamine/mTORC1 pathway. (**A** and **B**) Extracellular acidification rate (ECAR) (**A**) and oxygen consumption rate (OCR) (**B**) in KPTCs, measured using the Seahorse XF system. Primary KPTCs were cultured for 48 hours under the following conditions: LG, LG with glucosamine (GlcN, 250 or 500 μM), with or without rapamycin (rapa, 5 nM). OCR was measured at baseline, following 1.5 μM oligomycin, 1 μM FCCP, and 0.5 μM antimycin/rotenone treatment (*n* = 5 per group). (**C** and **D**) Relative abundance of ^13^C-labeled glucose and glycolytic intermediates (**C**) and tricarboxylic acid (TCA) cycle metabolites (**D**) in KPTCs. Primary KPTCs were cultured for 48 hours at LG, HG, or HG with rapamycin (rapa, 10 nM) (*n* = 4 per group). (**E**) NADH/NAD^+^ ratio, NAD^+^ levels (middle), and NADH levels (right) in KPTCs. Throughout the figure, data were analyzed by 1-way ANOVA. *P < 0.05; **P < 0.01.

In contrast, chronic exposure of KPTCs to HG or low-dose glucosamine (HG equivalent) increased glycolysis and basal and ATP-linked respiration, without affecting proton leak (Figure 5, A and B). Rapamycin prevented glucosamine stimulation of glycolysis and inhibited mitochondrial activity (Figure 5B). Metabolomics after ^13^C_6_-glucose labeling showed that exposure to HG increased the levels of labeled glucose and downstream glycolytic metabolites, including G6P, F6P, DHAP/G3P, and PEP, whereas the levels of pyruvate and lactate were unchanged, and certain TCA cycle intermediates (α-ketoglutarate and succinate) were reduced (Figure 5, C and D), suggesting dysregulation of glycolysis and glucose oxidation. Rapamycin markedly decreased G6P, F6P, pyruvate, and lactate, as well as glucose-derived acetyl CoA and malate, indicating inhibition of glycolysis and of glucose oxidation. Consistently, the levels of all glucose-derived AAs (serine, glycine, asparagine, aspartate, glutamate, and proline) were reduced (Supplemental Figure 5A). Treatment with rapamycin increased the levels of free fatty acids (FFAs) and glycerol, suggesting breakdown of triacylglycerols, along with increased availability of FFAs for energy production (Supplemental Figure 5B). Strikingly, treatment with rapamycin decreased NAD^+^ levels along with an increased NADH/NAD^+^ ratio (Figure 5E). A decreased NAD^+^ pool inhibits metabolic pathways, including glycolysis and the TCA cycle, resulting in decreased metabolic activity. Collectively, our findings suggest that inhibition of mTORC1 in KPTCs induces a protective metabolic reprogramming to manage the increased workload imposed by hyperglycemia. This adaptive shift is characterized by an inhibition of glycolysis and glucose oxidation, an enhanced reliance on fatty acid metabolism, and an overall reduction in mitochondrial work.

### Glucosamine regulation of mTORC1, insulin secretion, and mitochondrial activity in β cells.

We further studied the link between the HBP, mTORC1 activation, and overall metabolic function in human islets that were incubated at LG or HG for 48 hours followed by metabolic labeling with ¹³C-glucose. Metabolomics analysis showed that the abundance of ^13^C-labeled glycolysis and TCA cycle metabolites increased (Supplemental Figure 6). The levels of ^13^C_6_-glucosamine and its downstream product, UDP-GlcNAc, were also increased (Figure 1L). Collectively, these findings indicate that chronic exposure to HG enhances glycolysis, glucose oxidation, and flux through the hexosamine pathway.

**Figure 6:**
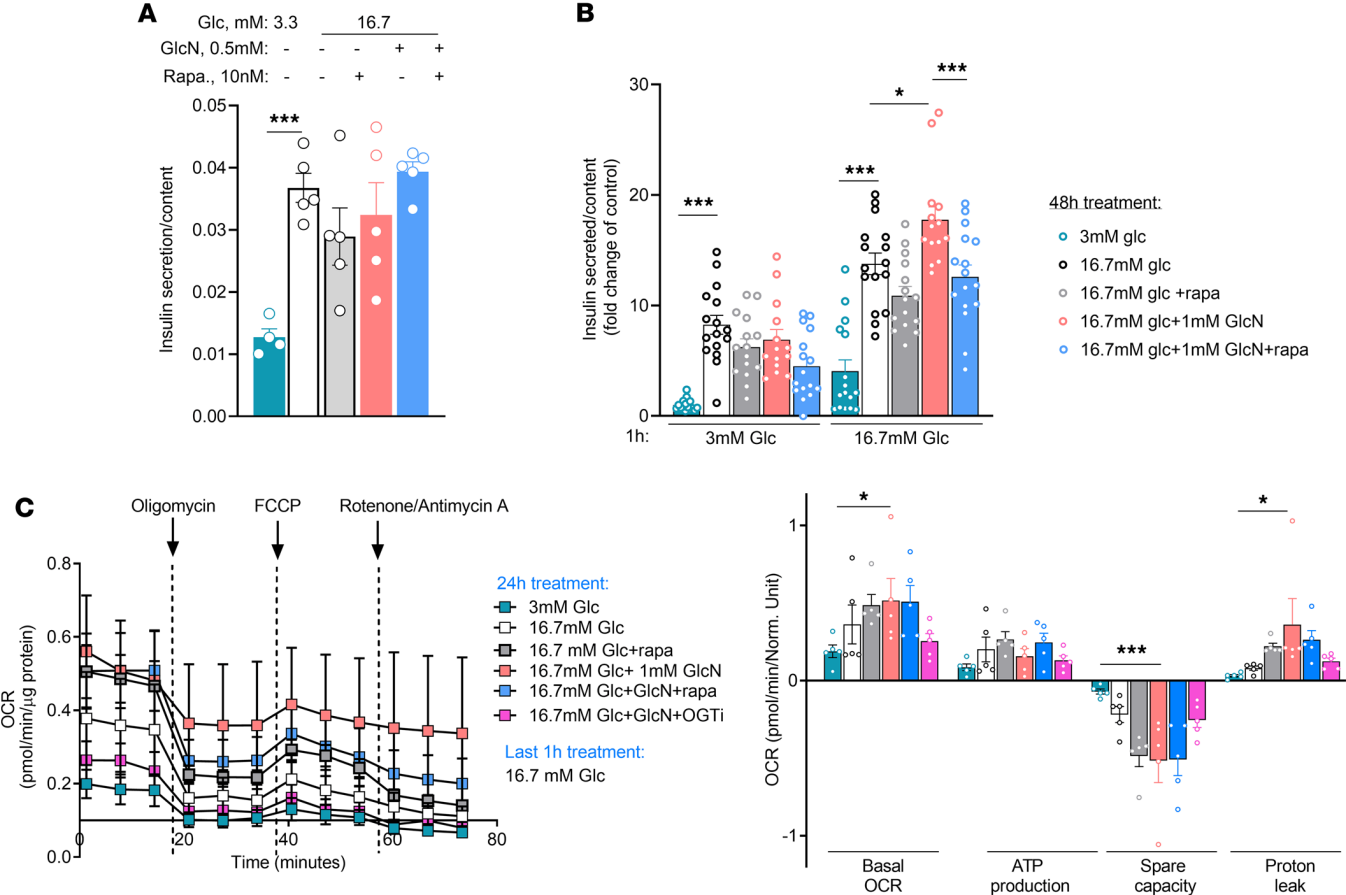
Glucosamine regulation of mTORC1, insulin secretion, and mitochondrial activity in β cells. (**A** and **B**) Mouse islets were incubated with LG or HG (3.3 mM or 16.7 mM glucose [Glc], respectively) with or without glucosamine (GlcN, 1 mM) and rapamycin (rapa, 10 nM) for 1 hour (**A**) and 48 hours (**B**). Insulin content and the fold change in insulin secretion normalized to islet insulin content are shown (5–9 biological replicates per group in 2 independent experiments). Data were analyzed by 1-way (**A**) and 2-way ANOVA (**B**). (**C**) Oxygen consumption rate (OCR) in dispersed mouse islets, measured using the Seahorse XF system. Islets were cultured for 48 hours under the following conditions: LG, HG with GlcN (1 mM), with or without rapa (10 nM) (*n* = 2, 5 biological replicates per group). Data were analyzed by 2-way ANOVA. *P < 0.05; ***P < 0.001.

Next, we studied the effects of glucosamine on insulin secretion. Acute exposure to glucosamine did not affect glucose-stimulated insulin secretion (Figure 6A). On the contrary, chronic islet incubation under HG with glucosamine markedly increased basal and glucose-stimulated insulin secretion (Figure 6B). Strikingly, treatment with rapamycin markedly reduced the amplification of insulin secretion by glucosamine (Figure 6B), indicating that this amplification is mTORC1 dependent.

We further studied the effect of glucosamine on mitochondrial function. Seahorse experiments showed that islets incubated at HG with glucosamine tended to increase basal oxygen consumption rate and ATP-linked respiration (not significant), along with increased proton leak and decreased mitochondrial spare capacity (Figure 6C). Treatment with OGTi, but not with rapamycin, attenuated these changes. Collectively, these findings show that in islets exposed to HG, glucosamine augments mitochondrial respiration in an OGT-dependent manner, along with development of mitochondrial dysfunction, evident by increased proton leak. Rapamycin inhibited the amplification of insulin secretion by glucosamine without affecting mitochondrial respiration, suggesting that mTORC1 modulates insulin secretion downstream of mitochondrial respiration/ATP generation.

### Effects of prolonged exposure to glucosamine on cellular stress and dysfunction.

**Figure 7:**
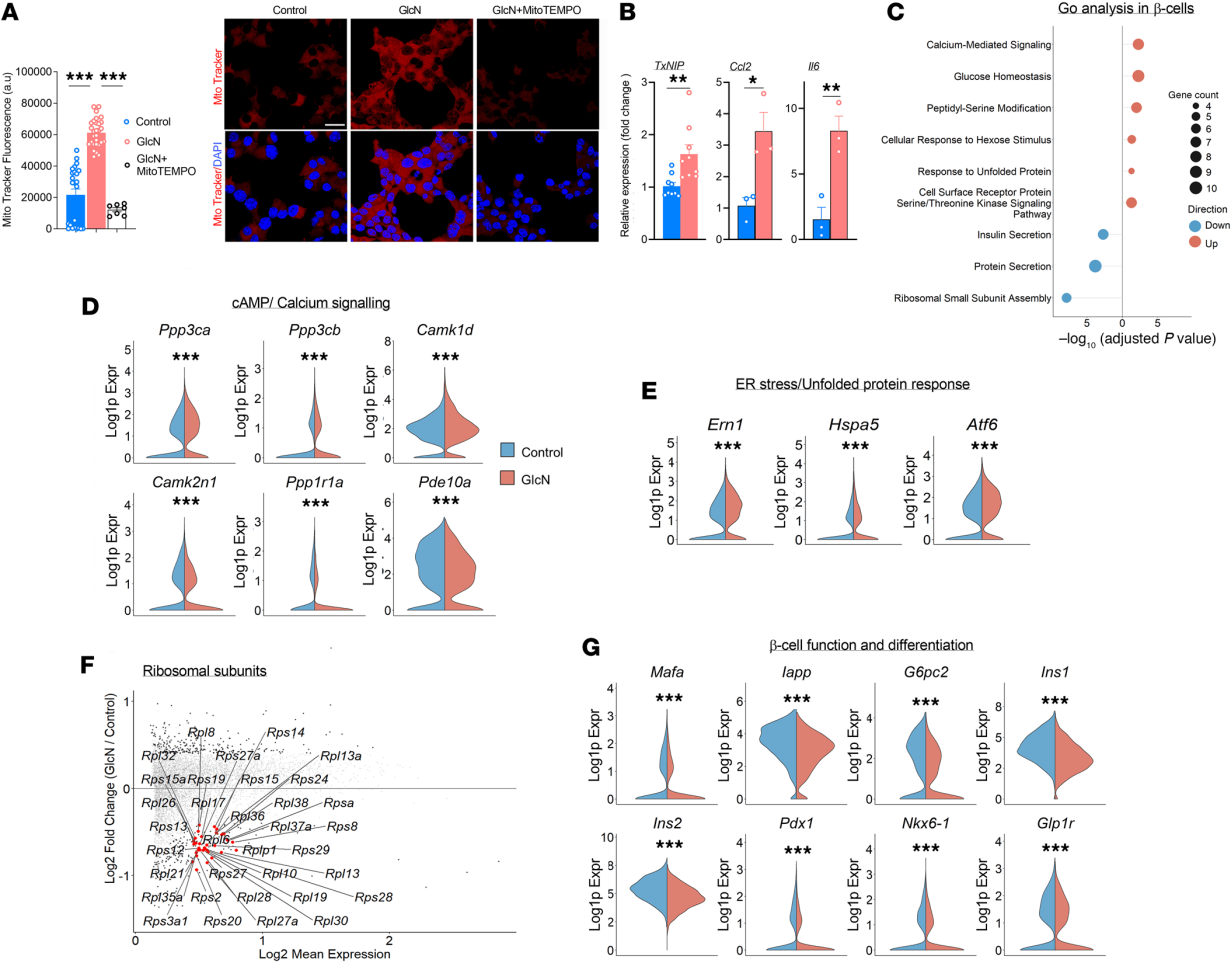
Effects of prolonged exposure to glucosamine on cellular stress and dysfunction. (**A**) MIN6 cells were treated with or without 1 mM glucosamine (GlcN) and MitoTEMPO, a specific scavenger of mitochondrial superoxide, for 24 hours. Cells were then stained with DAPI and MitoTracker Red CMXRos to assess mitochondrial membrane potential. Quantification of MitoTracker Red CMXRos fluorescence intensity and representative immunofluorescence images are shown (*n* = 3 per group). Data were analyzed by 1-way ANOVA. Original magnification, ×60. Scale bar: 20 μm. (**B**) Quantitative RT-PCR analysis of pro-oxidant (*Txnip*) and proinflammatory (*Ccl2*, *Il6*) gene expression in MIN6 cells following treatment with 1 mM GlcN for 24 hours (*n* = 3–9 per group). Data were analyzed by 1-sample Student’s *t* test. (**C**) Gene Ontology (GO) enrichment analysis of differentially expressed genes via hypergeometric testing. Dot size is proportional to the number of genes in each category, and color indicates net upregulation (orange) or downregulation (blue) of the pathway. (**D**, **E**, and **G**) Violin plots comparing control islets (blue) and GlcN-treated islets (orange) for gene expression associated with (**D**) cAMP and calcium signaling, (**E**) ER stress, and (**G**) β cell identity and function. (**F**) MA plot showing GlcN’s effect on the expression of genes encoding ribosomal subunits (*n* = 3 per group). Data were analyzed by the Wilcoxon rank-sum test.

Hyperglycemia and glucosamine enhanced insulin secretion, mitochondrial respiration, and glucose metabolism, indicating increased β cell workload, which could eventually lead to cellular stress and dysfunction. Consistently, treatment with glucosamine increased mitochondrial ROS generation in MIN6 cells (Figure 7A), along with increased expression of *Txnip*, a central mediator of oxidative stress and inflammation in islets (47), as well as the proinflammatory genes *Ccl2* and *Il6* (Figure 7B).

Next, we employed analyses of the transcriptome (snRNA-seq) of β cells to clarify the mechanisms underlying the glucosamine effects. Gene expression analysis showed that treatment with glucosamine enriched the expression of genes associated with calcium and cAMP signaling, cytoskeleton organization, ER stress, and β cell function and differentiation (Figure 7C). Treatment with glucosamine increased the expression of *Ppp3ca* and *Ppp3cb*, which encode the catalytic subunits (α and β, respectively) of calcineurin, a Ca^2+^/calmodulin-dependent serine/threonine phosphatase that is essential for Ca^2+^-dependent signal transduction (48) (Figure 7D and Supplemental Figure 7A). The expression of Ca^2+^/calmodulin-activated kinase 1d (*Camk1d*) was also upregulated. Both calcineurin- and Ca^2+^/calmodulin-activated kinases are central regulators of insulin secretion, ER calcium homeostasis, and cellular stress adaptation, and play a role in diabetes pathophysiology (49). In contrast, the expression of Ca^2+^/calmodulin-dependent protein kinase II inhibitor 1 (*CamK2n1*) that acts as an endogenous inhibitor of CaMKII and of protein phosphatase 1 regulatory subunit 1A (*Ppp1r1a*) was downregulated. PPP1R1A acts as a cAMP/PKA-responsive inhibitor of protein phosphatase 1 (PP1), and its expression in β cells is required for optimal glucose-stimulated insulin secretion, biosynthesis, and for maintenance of β cell identity (50, 51).

Overall, the expression of central genes regulating calcium fluxes and signaling were altered, probably leading to β cell dysfunction, ER stress, and dedifferentiation. Consistently, the expression of key genes involved in the ER stress response/unfolded protein response (UPR), including *Hspa5* (BIP), *Ern1* (Ire1a), and *Atf6*, was increased (Figure 7E and Supplemental Figure 7B); this was accompanied by decreased expression of multiple ribosome subunits, probably as an adaptive response to ER stress (Figure 7F). In addition, genes involved in mitochondria function, redox state (Supplemental Figure 7C), and cytoskeleton remodeling (Supplemental Figure 7D) were upregulated. Strikingly, key genes regulating β cell function and differentiation, including *Pdx1*, *Nkx6.1*, *Mafa*, *Glp1r*, and *Ins1/2*, were downregulated (Figure 7G and Supplemental Figure 7E).

In summary, prolonged exposure to glucosamine stimulates mTORC1, along with dysregulation of mitochondrial function and the expression of key genes regulating calcium homeostasis, resulting in oxidative stress, ER stress, and β cell dysfunction, possibly via dedifferentiation.

### Effects of inhibiting β cell mTORC1 activity in diabetes.

**Figure 8:**
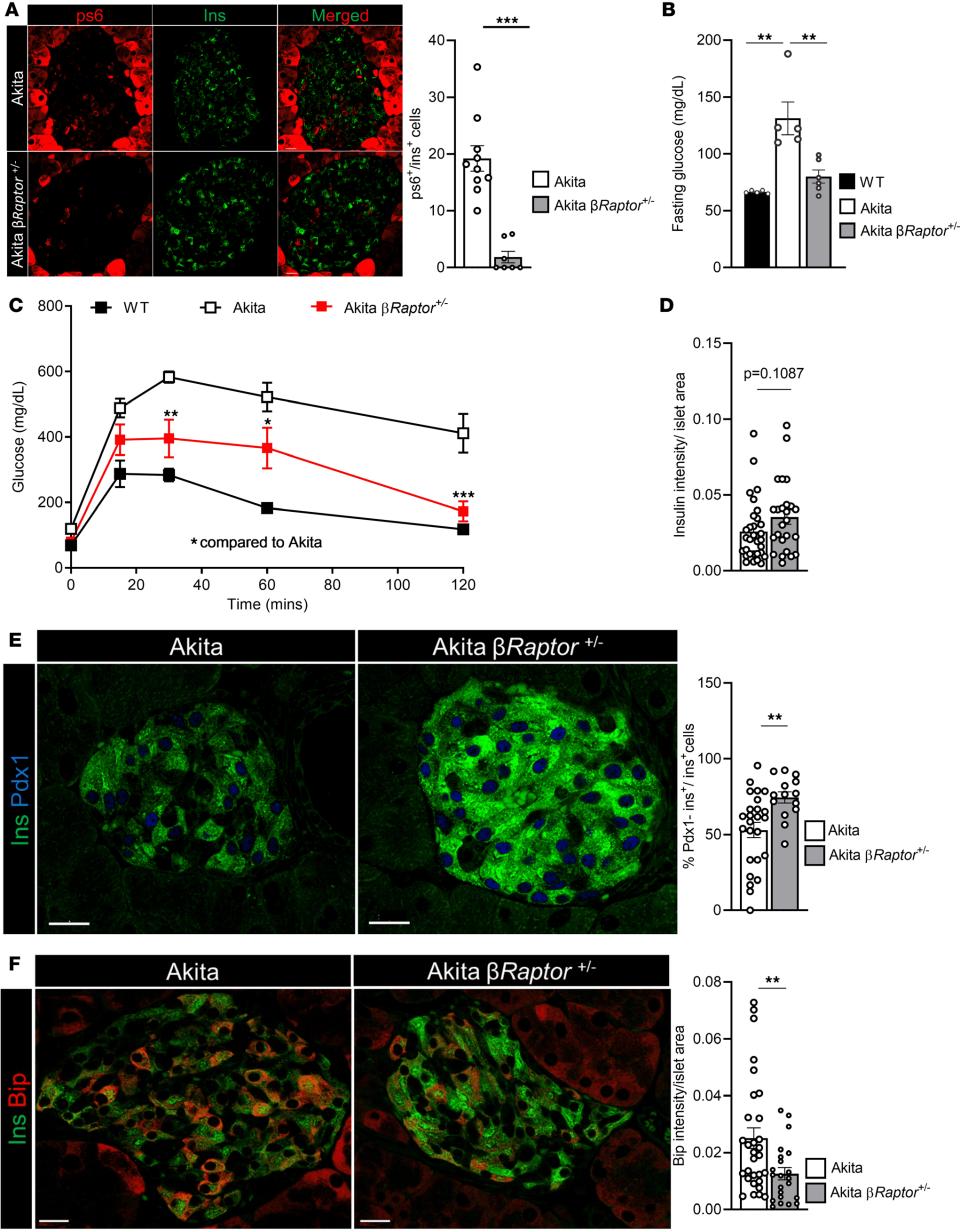
mTORC1 inhibition in Akita β cells improves diabetes and β cell function. Akita; *MIP-CreER;Raptor^+/fl^* mice were injected with tamoxifen to generate conditional heterozygous *Raptor* KO in β cells. (**A**) mTORC1 activity in β cells. Pancreatic sections from Akita mice and Akita; β*Raptor^+/−^* mice immunostained for p-S6 and insulin; quantification of p-S6^+^ β cells is shown (*n* = 1–2 mice; 189–439 cells analyzed per group). Each dot represents the percentage of positive cells. Data were analyzed by Student’s *t* test. (**B**–**F**) Effects on glucose tolerance and insulin production were assessed 14 days after tamoxifen injection. (**B**) Fasting blood glucose levels in WT, Akita, and Akita; β*Raptor*^+/−^ mice (*n* = 5 per group). Data were analyzed by 1-way ANOVA. (**C**) IPGTT (*n* = 3–4 mice per group). Data were analyzed by 2-way ANOVA. (**D**) Insulin intensity per islet. (**E**) Percentage of PDX-1^+^ β cells, and (**F**) BIP intensity per islet area in sections from Akita and Akita; β*Raptor*^+/−^ mice. **D**–**F**, *n* = 3–4 per group. Data were analyzed by Student’s *t* test. Pancreatic sections were immunostained for insulin and PDX-1 (743–1755 β cells counted) or BIP (330–677 β cells counted). *P < 0.05; **P < 0.01; ***P < 0.001.

We have previously shown that inhibiting mTORC1 by heterozygous KO of *Raptor* in KPTCs prevents kidney fibrosis in the Akita model of DKD, in which kidney dysfunction develops due to prolonged exposure to hyperglycemia (12). Here, we further studied the role of mTORC1 in mediating β cell dysfunction in this ER stress–induced neonatal diabetes model. mTORC1 plays a role in disease pathophysiology; it is inhibited during the neonatal period, thereby preventing β cell expansion in early life, followed by reactivation during adulthood in parallel with the development of hyperglycemia (15). We generated heterozygous β cell *Raptor*-KO (β*Raptor*^+/–^) Akita mice and confirmed successful recombination (Figure 8A); we then assessed the effects on diabetes and β cell function (Figure 8, B–F). Fasting blood glucose levels were lower in β*Raptor*^+/–^ Akita mice when compared with their β*Raptor*^+/+^ littermates (Figure 8B). Intraperitoneal glucose tolerance test (IPGTT) showed that glucose tolerance was improved (Figure 8C), along with a tendency to increased islet insulin content (*P* = 0.1) (Figure 8D). Inhibition of mTORC1 increased the protein level of PDX-1 (Figure 8E), which plays an important role in the regulation of insulin synthesis in adult β cells. The expression of the ER stress marker BIP was decreased (Figure 8F). Collectively, these findings suggest that inhibiting mTORC1 improved diabetes and β cell function by reducing ER stress.

**Figure 9:**
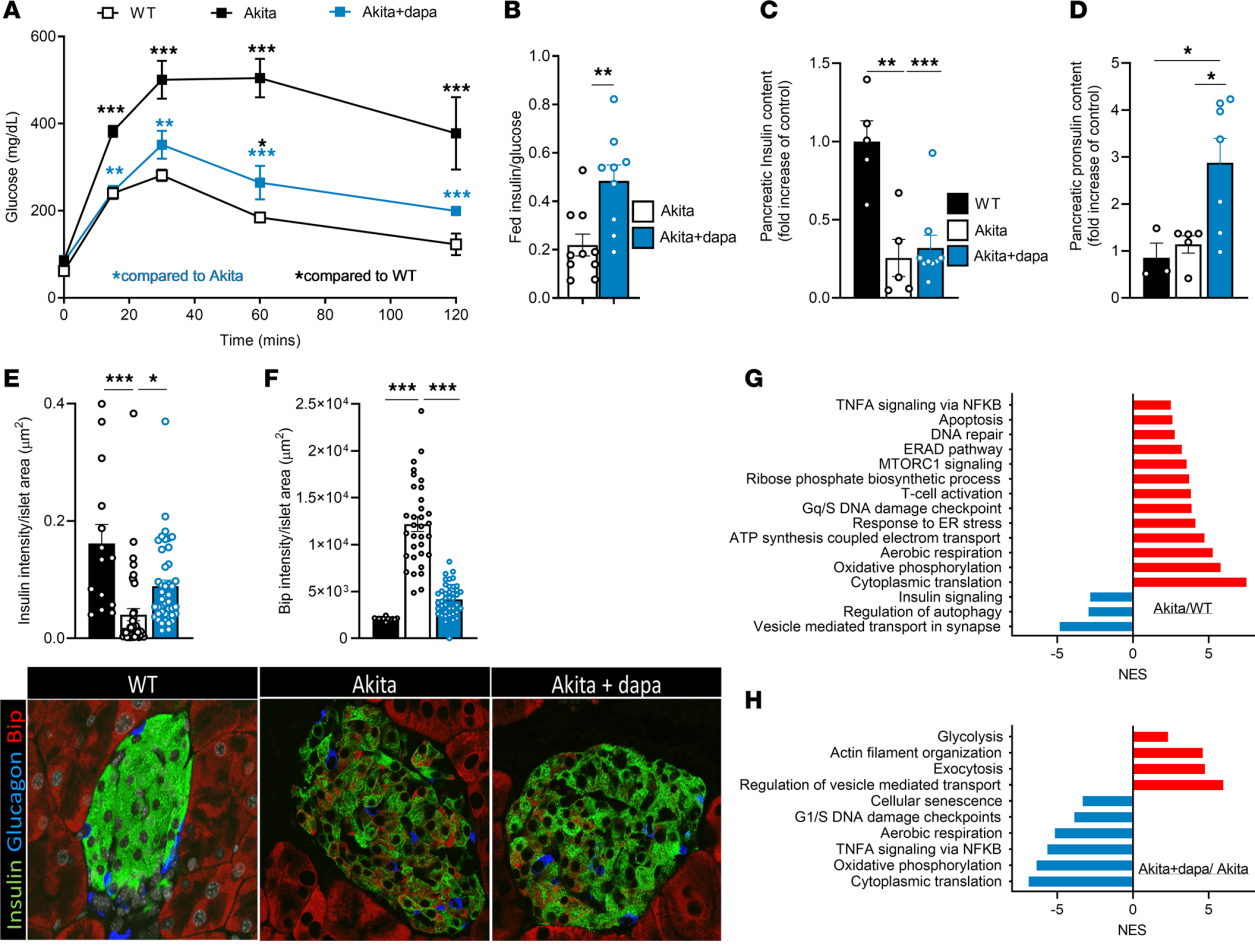
Dapagliflozin improves diabetes and β cell function, along with decreased ER stress and β cell inflammatory signature in Akita mice. Six-week-old Akita mice were treated with or without dapagliflozin (10 mg/kg/day in drinking water) for 6 weeks and compared to WT mice. (**A**) IPGTT (1.3 g/kg) (*n* = 3–6 mice per group). Data were analyzed by 1-way ANOVA. (**B**) Fed plasma insulin normalized to glucose (*n* = 8–10). Data were analyzed by Student’s *t* test. (**C** and **D**) Pancreatic insulin and proinsulin content measured by ELISA (*n* = 3–8). Data were analyzed by 1-way ANOVA. (**E** and **F**) Pancreatic sections stained for insulin and BIP. Quantification of insulin and BIP signal intensity per islet area is shown (*n* = 2–3 mice per group, 10 islets quantified per group). Data were analyzed by 2-way ANOVA. (**G** and **H**) Comparative transcriptome analysis based on RNA-seq data of islets from Akita mice treated with and without dapagliflozin and WT controls. (**G**) Akita compared to WT islets. (**H**) Akita mice treated with dapagliflozin compared to Akita controls. Differentially regulated pathways are shown (*n* = 3 per group, each repeat includes a pool of islets from 2–3 mice).

The SGLT2i dapagliflozin effectively inhibited the glucosamine/mTORC1 pathway, evident by decreased glucosamine levels, along with inhibition of mTORC1. As previously shown (15), treatment with dapagliflozin decreased mTORC1 activity in Akita β cells (Supplemental Figure 8A). We treated Akita mice with dapagliflozin for 2–6 weeks, followed by washout leading to complete elimination of the drug (confirmed by LCMS) (Supplemental Figure 8B); glucose tolerance was improved (Figure 9A). Plasma insulin levels were increased in Akita mice treated with dapagliflozin compared with Akita controls (Figure 9B). β Cell area was decreased in Akita mice and was not affected by treatment with dapagliflozin (Supplemental Figure 8C). In Akita mice, pancreatic insulin content was decreased by approximately 90%, whereas treatment with dapagliflozin induced 2- and 3-fold increases in insulin and proinsulin content, respectively, indicating partial restoration of insulin production (Figure 9, C–E). Treatment with dapagliflozin did not affect the proinsulin/insulin ratio (Supplemental Figure 8D), suggesting that it mainly affected (pro)insulin synthesis without affecting the efficiency of proinsulin processing. PDX-1 and NKX6.1 protein levels were slightly decreased in Akita β cells, whereas treatment with dapagliflozin restored the expression of these transcription factors, which are required for adult β cell function (Supplemental Figure 8, E and F). Treatment with dapagliflozin decreased the expression of the ER stress marker BIP, along with increased pancreatic (pro)insulin content (Figure 9, C–F), indicating that dapagliflozin enhanced insulin production by alleviating ER stress.

We performed bulk RNA-seq on Akita islets treated with and without dapagliflozin for 2 weeks and compared gene expression to WT islets. GSEA showed that genes involved in metabolism (oxidative phosphorylation/aerobic respiration/ATP synthesis–coupled electron transport), ER stress, DNA damage repair, apoptosis, inflammation, and mTORC1 signaling were enriched in Akita compared with WT islets, whereas genes associated with autophagy were downregulated (Figure 9G). Treatment with dapagliflozin enriched the expression of genes regulating exocytosis and actin filament network organization, whereas genes involved in metabolism (oxidative phosphorylation/aerobic respiration) and translation, along with DNA damage response, inflammation, and senescence were downregulated (Figure 9H). The expression of several genes involved in the ER stress response, including *Sec61b*, which is involved in ER protein translocation, *Cops5*, a positive regulator of E3 ubiquitin ligases that are involved in the degradation of cyclin inhibitor kinase CDKN1B/p27Kip1, *Ufc1*, *Ude2J2*, *Ube2k* (ubiquitination), and *Psmc6* (proteasome function) were increased in Akita islets and decreased in response to treatment with dapagliflozin (Supplemental Figure 8G). In addition, the protein disulfide isomerase *Erp44* that promotes protein folding in the ER, *Ptpn2*, a phosphatase that regulates cell growth, proliferation, and differentiation, and *Xbp1* that plays a role in the regulation of the UPR and of the immune system were upregulated in Akita islets and decreased with dapagliflozin treatment (Supplemental Figure 8G). Interestingly, the expression of several genes that were not affected by diabetes in Akita mice were increased by dapagliflozin, e.g. *Edem3*, which regulates the degradation of misfolded glycoproteins in the ER, and the phospholipases *Pla2g6* and *Bhlha15*, which are involved in several processes, including cellular response to glucose starvation and UPR (Supplemental Figure 8G).

The expression of proinflammatory genes, including *Tnfa* and *Il6* and the chemokines *Cxcl1* and *Ccl2*, were increased in Akita islets and decreased with dapagliflozin treatment (Supplemental Figure 8H). Key regulators of the cell cycle, including *Cdkn1a* (p21, a cyclin-dependent kinase inhibitor involved in senescence), as well as *Cdkn1b* and *Ccna2* were increased in Akita and decreased with dapagliflozin treatment (Supplemental Figure 8I). Several genes involved in calcium signaling and secretory granule trafficking and fusion were downregulated in Akita islets and rescued by dapagliflozin treatment (Supplemental Figure 8J). In summary, treatment with an SGLT2i induces sustained improvement of diabetes and β cell function, along with modulation of the ER stress response, despite persistent proinsulin misfolding. Improved β cell function is explained by recovery of proinsulin synthesis and increased expression of genes regulating exocytosis, along with downregulation of the proinflammatory genes.

The genetic pathways that were altered in diabetic Akita β cells partially mimicked those induced by glucosamine. This aligns with our finding that intracellular glucosamine levels were elevated in Akita islets and normalized with dapagliflozin treatment (Figure 1A). Consistently, the beneficial effects of dapagliflozin resembled those observed by genetic inhibition of mTORC1 in diabetic β cells. Therefore, we suggest that the glucosamine/mTORC1 pathway contributes to glucotoxicity in diabetes. SGLT2is may exert their beneficial effects in different organs through inhibition of the glucosamine/mTORC1 pathway.

## Discussion

Our findings demonstrate that glucosamine transduces mTORC1 activation in response to chronic hyperglycemia and contributes to glucotoxic effects in diabetes. Several lines of evidence support this conclusion: (a) glucosamine levels are increased in diabetes, whereas glucose-lowering treatments (insulin and SGLT2is) reduce glucosamine across multiple tissues; (b) the glucosamine metabolite GlcNAc-1-P is increased in the plasma of individuals with IFG and T2D and inversely correlates with β cell function; (c) tissue glucosamine levels closely correlate with tissue glucose levels; (d) incubation of KPTCs and islets in HG induces de novo glucosamine generation; (e) treatment of KPTCs, islets, and β cells with glucosamine at concentrations comparable to those measured in HG-exposed tissues activates mTORC1; (f) glucosamine enters the hexosamine pathway and stimulates mTORC1 in an OGT/OGA-dependent manner; (g) glucosamine enhances mitochondrial respiration in islets and KPTCs (in β cells, glucosamine dysregulates the expression of key genes involved in calcium homeostasis, induces ER stress, and promotes β cell dedifferentiation, thereby mimicking β cell dysfunction seen in diabetes); and (h) inhibition of mTORC1 by generating a heterozygous *Raptor* loss-of-function mutation in β cells ameliorates diabetes and reduces β cell stress and inflammation.

Previous studies proposed that upstream glycolytic intermediates and enzymes may act as sensors linking glucose metabolism to mTORC1 activity in various cell types. For example, in mouse embryonic fibroblasts, F1,6-BP binds aldolase, enabling interaction with the lysosomal v-ATPase complex, thereby activating mTORC1 and inhibiting AMPK (52–54). Others reported that PFK and PFKFB3 associate with the RagB GTPase–Ragulator complex at the lysosome (55). The Sabatini group identified DHAP as an mTORC1 activator in HEK cells (56), whereas other studies showed that G3P, also generated by aldolase, prevents GAPDH binding to Rheb, thereby inhibiting mTORC1 (57). Conversely, in INS-1 β cells, knockdown or pharmacologic inhibition of GAPDH activated, rather than suppressed, mTORC1, suggesting that the relevant mTORC1-stimulating metabolite(s) lies upstream of GAPDH (9). While glucose-mTORC1 coupling may be cell-type specific, previous efforts relied on genetic or pharmacologic perturbation of glycolytic enzymes, which produces broad effects on metabolism, energy generation, and redox state, all of which can influence mTORC1 activity.

To elucidate underlying mechanisms, we performed in vivo ^13^C_6_-glucose labeling in normoglycemic and diabetic animals, with or without glucose-lowering therapies, followed by broad, unbiased metabolomics, without manipulating glycolytic enzymes. Unexpectedly, glucosamine, but not other known mTORC1-stimulating metabolites such as branched-chain AAs, methionine, *S*-adenosylmethionine, or glycolytic intermediates, showed tight correlation with tissue glucose. Glucosamine was elevated in kidney cortex, liver, and heart from hyperglycemic animals, and in KPTCs and islets exposed to HG. We further show that glucosamine is synthesized de novo through glucose amination, likely via amine transfer from glutamine (and possibly additional amino acids). Because glucosamine is not abundant in most foods, its generation is largely independent of dietary intake, making it a plausible intracellular sensor of glucose metabolism.

In mammals, glucosamine is predominantly present as GlcN-6-P, produced from F6P by GFAT, the first committed step of the hexosamine pathway (22, 23). Here, we describe what we believe is a novel, non-canonical pathway that predominates in cells exposed to HG. Consistent with previous reports (25), we found that glucosamine phosphorylation to GlcN-6-P is mediated by hexokinases. Inhibition of hexokinase/glucokinase with 2-DG decreased GlcN-6-P levels and suppressed glucosamine-induced mTORC1 activation in KPTCs.

O-GlcNAcylation is a dynamic form of glycosylation in which GlcNAc cycles on and off serine and threonine residues. Since its discovery 40 years ago (26), more than 16,000 O-GlcNAc–modified proteins have been identified, spanning transcription, translation, nutrient sensing, immunity, cell cycle, circadian regulation, and cell signaling (58). In HEK cells, HG and glucosamine stimulate mTORC1 via O-GlcNAcylation of Raptor at threonine 700, promoting activity under nutrient-replete conditions when AMPK is inactive (35). Glucose availability also regulates mTORC1 via the intracellular leucine sensor LARS1 (59); glucose starvation induces O-GlcNAcylation of LARS1, inhibiting its interaction with RagD and decreasing its affinity for leucine, thereby integrating glucose and AA signals. Our phosphoproteome and transcriptome analyses showed that glucosamine altered the phosphorylation and gene expression of multiple regulators of mTORC1, including RAF1 (MAP/ERK signaling), PIKFYVE (mTOR attachment to the lysosome), and AKAP13, which modulates mTORC1 activity in response to cAMP/PKA. In addition, glucosamine increased the expression of mTOR itself, as well as genes involved in growth factor signaling to mTORC1, e.g., *Igf1r*, *Pikfye*, and *Fnip1* (folliculin-interacting peptide), and *Lars1*. These changes may explain the sustained activation of mTORC1 by glucosamine.

Both O-GlcNAcylation and mTORC1 activity oscillate with feeding-fasting cycles (60, 61). Chronic hyperglycemia may disrupt this rhythmicity, increasing cellular workload and contributing to diabetes and its complications. Our findings support this model; prolonged HG or glucosamine exposure induced sustained mTORC1 activation in KPTCs and β cells, accompanied by increased basal and ATP-linked mitochondrial respiration. In KPTCs, mTORC1 inhibition by rapamycin triggered metabolic reprogramming, including reduced glycolysis and oxidative phosphorylation, and a substrate shift from glucose to fatty acids, evidenced by decreased glucose oxidation and glycogenic AA synthesis, and increased FFAs and glycerol.

In β cells, chronic exposure to HG and glucosamine increased glycolysis, glucose oxidation, mitochondrial respiration, and insulin secretion, the latter being inhibited by rapamycin. Glucosamine-stimulated β cell respiration was associated with oxidative stress and increased expression of the key mediators of ER stress, along with a robust decrease in β cell genes that ultimately impair β cell function and differentiation.

In diabetes, reducing cellular workload may alleviate oxidative stress, ER stress, and inflammation, but excessive inhibition could be detrimental, particularly in β cells, which must sustain insulin output. Given the dual (“Yin-Yang”) roles of mTORC1, moderate, fine-tuned inhibition may be optimal.

Indeed, we previously showed that partial mTORC1 inhibition via heterozygous *Raptor* deletion in KPTCs prevented DKD in Akita mice (12). We used a similar strategy to study the effects of moderate mTORC1 reduction in ER-stressed β cells in diabetes. Akita mice develop diabetes due to a proinsulin missense mutation that causes irreversible misfolding and lifelong β cell ER stress, modeling the neonatal diabetes syndrome mutant-insulin diabetes of the young (MIDI) (62). mTORC1 is suppressed during the neonatal period due to ER stress, impairing β cell expansion and differentiation and reducing insulin secretion (15). In Akita mice, hyperglycemia after weaning is associated with mTORC1 reactivation (15). Remarkably, heterozygous β cell *Raptor* deletion in adult male Akita mice improved diabetes and partially preserved insulin secretion.

Paradoxically, dietary D-glucosamine supplementation extends lifespan in nematodes and mice (63) and ameliorates insulin resistance and inflammation in metabolic dysfunction–associated steatotic liver disease (MASLD) models (64). Oral glucosamine inhibits hexokinases and glycolysis and activates AMPK, mimicking caloric restriction similarly to 2-DG (65). We hypothesize that exogenous glucosamine competes with glucose for transporter binding and inhibits hexokinase/glucokinase, thereby suppressing both glycolysis and conversion of glucosamine to GlcN-6-P.

In contrast, our findings underscore the importance of intracellular glucosamine synthesis and its metabolism through the hexosamine pathway in mediating hyperglycemia-induced mTORC1 activation and glucotoxicity. We propose that glucosamine functions primarily as a rheostat for intracellular glucose availability, thereby regulating mTORC1 activity and mediating glucotoxicity.

At the translational level, we show that the SGLT2i dapagliflozin, widely used for diabetes, kidney disease, and heart failure, reduces glucosamine levels and inhibits mTORC1. This was associated with sustained improvement in diabetes and β cell function, attenuation of ER stress responses, and decreased inflammatory gene expression. Chronic kidney injury in diabetes and other kidney diseases enhances glucose uptake in KPTCs, promoting glycolysis, increased O-GlcNAcylation, and mTORC1 activation (33, 66–68). Hyperactivation of the hexosamine pathway also contributes to pathological cardiac hypertrophy and heart failure via persistent mTORC1 activation (69). SGLT2is consistently improve DKD, non-DKDs, and heart failure (70–72). We propose that inhibition of the glucosamine/mTORC1 pathway may partially mediate these robust renal and cardiac benefits. Furthermore, SGLT2is may provide durable improvement in β cell function even under severe ER stress, thereby slowing diabetes progression.

### Study limitations.

Our data suggest that in diabetes, glucosamine is a central mediator of mTORC1 activation in different tissues. However, we can not exclude the possibility that AAs and/or other glycolytic intermediates regulate mTORC1 in a tissue- or context-dependent manner. Hyperglycemia may induce cellular stress and tissue dysfunction through additional mechanisms independent of glucosamine/mTORC1 signaling. Moreover, OGT regulates O-GlcNAcylation of numerous proteins, which may contribute to glucotoxicity. Nonetheless, we show that glucosamine is consistently increased in multiple diabetic tissues and that glucosamine activates mTORC1 in an OGT-dependent manner. Partial mTORC1 inhibition in adult diabetic β cells and KPTCs improved diabetes, β cell function, and DKD, supporting a role for this pathway in disease pathogenesis. However, future studies are warranted to extend these findings to other models of diabetes (e.g. obesity-associated T2D) and to further dissect the precise molecular interplay between O-GlcNAcylation and specific components of the mTORC1 signaling complex.

In summary, we identify endogenous glucosamine as a central mediator of mTORC1 activation by hyperglycemia. Glucosamine alters the phosphorylation and expression of upstream regulators of mTORC1, leading to its sustained activation, which contributes to β cell dysfunction in diabetes. SGLT2 inhibition reduces glucosamine levels, suppresses mTORC1, improves β cell function, and may prevent kidney disease in part through inhibition of the glucosamine/mTORC1 pathway.

## Methods

### Sex as a biological variable.

Only male Akita mice and transgenic heterozygous *Raptor*-KO Akita mice were studied because female Akita mice develop only mild diabetes and to avoid leakiness of the CreER system due to activation of Cre by circulating estrogen in female mice. The findings are expected to be relevant to the mechanisms of glucotoxicity in both males and females.

### Animals.

Mice were housed in the Hebrew University animal care unit with 12-hour light/dark cycles and fed standard chow diet and water ad libitum. Eight-week-old male WT C57BL/6 mice fed regular chow and diabetic male Akita (Ins2WT/C96Y) mice (The Jackson Laboratory) were treated with dapagliflozin (10 mg/kg/day in drinking water; Forxiga, AstraZeneca) for 1 week. When specified, Akita mice received a subcutaneous injection of insulin (2–4 U/day, degludec, Novo Nordisk) for 7 days. *MIP-Cre^ER^* mice were provided by Yuval Dor (The Hebrew University) and *Raptor^fl/fl^* (*Rptor*^tm1.1Dmsa^) mice were obtained from The Jackson Laboratory (strain 013188). Mice were crossed to generate *MIP-CreER^+/–^;Raptor^fl/+^* mice on the Akita background. *Raptor* deletion was induced by subcutaneous injection of 6-week-old transgenic mice with 2 daily doses of 8 mg tamoxifen (20 mg/mL in corn oil; Sigma-Aldrich). Littermate control mice (*Akita;* β*Raptor^+/–^* mice) included Akita mice bearing *MIP-CreER^+/–^;Raptor^fl/+^* that were injected with oil, or *Raptor^fl/+^* Akita mice injected with tamoxifen. When specified, Akita mice were treated with subcutaneous injection of degludec insulin (2–4 U/day; Novo Nordisk) or with dapagliflozin (10 mg/kg/day in drinking water; AstraZeneca) for 5 days followed by intraperitoneal injection of ^13^C_6_-D-glucose (1 mg/g; Sigma-Aldrich). After plasma was drawn, mice were euthanized by cervical dislocation and kidneys, liver, heart, pancreases, or islets were harvested for further analyses.

Detailed information on the in vivo metabolic tests in mice, metabolomics analyses, primary islet and KPTC isolation and cell line culture, Seahorse analyses, Western blotting, immunofluorescence, mitochondrial labeling with MitoTracker Red CMXRos, flow cytometry, RNA extraction, quantitative real-time PCR, RNA-seq data processing and analysis, and phosphoproteomics is provided in the Supplemental Methods.

### Data availability.

The sequencing data generated in this study have been deposited in the NCBI Gene Expression Omnibus (GEO). The snRNA-seq data from islets treated with glucosamine are accessible under accession number GSE325252. The bulk RNA-seq data from islets of WT and Akita mice treated with dapagliflozin are available under accession number GSE284078. The raw metabolomics and phosphoproteomic data generated during this study are available from the corresponding author upon request.

### Statistics.

Data are expressed as the mean ± SEM, with statistical significance defined as a *P* value of less than 0.05. For general statistical analyses, 2-group comparisons were performed using unpaired, 2-tailed Student’s *t* tests unless specified otherwise, while multiple-group comparisons utilized 1-way or 2-way ANOVA with Bonferroni’s post hoc test. Pearson’s correlation coefficient (*r*) was determined via linear regression. All analyses were performed using Prism 8.0.2 (GraphPad Software Inc.). For RNA-seq experiments, analyses were conducted in R (version 4.5.0, https://cran.r-project.org/src/base/R-4/R-4.5.0.tar.gz); differentially expressed genes were identified using the Wilcoxon rank-sum test with Bonferroni’s correction, and pathway enrichment was assessed via a Gene Ontology (GO) analysis using a hypergeometric test with a Benjamini-Hochberg correction for the false discovery rate.

### Study approval.

The present studies in animals were reviewed and approved by the Institutional Animal Care and Use Committee of The Hebrew University of Jerusalem. Human islets were obtained from Prodo, which holds an independent institutional review board exemption from Western Institutional Review Board for the “Use of Human Islets for Research” stating no approval from human subjects is required since all pancreases were from cadaver organ donors less than 45 years old (CFR 46.102h).

## Author contributions

GL, ED, YR, and AKL conceived the study. GL, YR, AKL, EZ, TC, ZT, FG, IA, and BA designed experiments. YR, AKL, TC, AN, IS, IA, BA, LG, and DK performed experiments and analyzed results. EC, LH, JT, RP, DA, ED, and EBM were involved in the analysis and interpretation of the data. GL, YR, and AKL wrote the manuscript.

## Conflict of interest

The authors have declared that no conflict of interest exists.

## Funding support

This work is the result of NIH funding, in whole or in part, and is subject to the NIH Public Access Policy. Through acceptance of this federal funding, the NIH has been given a right to make the work publicly available in PubMed Central.

- National Institute of Diabetes and Digestive and Kidney Diseases grants R01-DK073716 (to EBM and GL), R01-DK132103 (to EBM), and R01-DK133183 (to EBM).
- US Department of Veterans Affairs Merit Award BX002728 (to EBM).
- European Foundation for the Study of Diabetes/Novo Nordisk A/S Programme for Diabetes Research in Europe (to GL).
- Israel Science Foundation grant ISF-398/20 (to GL).
- Israel Science Foundation–Juvenile Diabetes Research Foundation grant ISF-2982/20 (to GL).

## Supplementary Material

Supplemental data

Unedited blot and gel images

Supplemental table 1

Supporting data values

## Version 1 05/22/2026

Electronic publication

## References

[1] Kahn SE (2021). The β cell in diabetes: integrating biomarkers with functional measures. Endocr Rev.

[2] Afkarian M (2016). Clinical manifestations of kidney disease among US adults with diabetes, 1988-2014. JAMA.

[3] Lytvyn Y (2020). The new biology of diabetic kidney disease-mechanisms and therapeutic implications. Endocr Rev.

[4] Weng J (2008). Effect of intensive insulin therapy on beta-cell function and glycaemic control in patients with newly diagnosed type 2 diabetes: a multicentre randomised parallel-group trial. Lancet.

[5] Diabetes C (2000). Retinopathy and nephropathy in patients with type 1 diabetes four years after a trial of intensive therapy. N Engl J Med.

[6] Glaser B, Cerasi E (1999). Early intensive insulin treatment for induction of long-term glycaemic control in type 2 diabetes. Diabetes Obes Metab.

[7] Ardestani A (2018). mTORC1 signaling: a double-edged sword in diabetic β cells. Cell Metab.

[8] Haythorne E (2022). Altered glycolysis triggers impaired mitochondrial metabolism and mTORC1 activation in diabetic β-cells. Nat Commun.

[9] Kim J, Guan KL (2019). mTOR as a central hub of nutrient signalling and cell growth. Nat Cell Biol.

[10] Liu GY, Sabatini DM (2020). mTOR at the nexus of nutrition, growth, ageing and disease. Nat Rev Mol Cell Biol.

[11] Goul C (2023). The molecular basis of nutrient sensing and signalling by mTORC1 in metabolism regulation and disease. Nat Rev Mol Cell Biol.

[12] Kogot-Levin A (2020). Proximal tubule mTORC1 is a central player in the pathophysiology of diabetic nephropathy and its correction by SGLT2 inhibitors. Cell Rep.

[13] Tomita I (2020). SGLT2 inhibition mediates protection from diabetic kidney disease by promoting ketone body-induced mTORC1 inhibition. Cell Metab.

[14] Riahi Y (2023). Hyperglucagonaemia in diabetes: altered amino acid metabolism triggers mTORC1 activation, which drives glucagon production. Diabetologia.

[15] Riahi Y (2018). Inhibition of mTORC1 by ER stress impairs neonatal β-cell expansion and predisposes to diabetes in the *Akita* mouse. Elife.

[16] Kogot-Levin A (2023). Mapping the metabolic reprogramming induced by sodium-glucose cotransporter 2 inhibition. JCI Insight.

[17] Rorsman P, Ashcroft FM (2018). Pancreatic β-cell electrical activity and insulin secretion: of mice and men. Physiol Rev.

[18] Gilbert RE (2017). Proximal tubulopathy: prime mover and key therapeutic target in diabetic kidney disease. Diabetes.

[19] Ahmad M (2022). Kidney proximal tubule GLUT2-more than meets the eye. Cells.

[20] Roseman S (2001). Reflections on glycobiology. J Biol Chem.

[21] Oikari S (2016). Hexosamine biosynthesis in keratinocytes: roles of GFAT and GNPDA enzymes in the maintenance of UDP-GlcNAc content and hyaluronan synthesis. Glycobiology.

[22] Nelson ZM (2024). Tools for investigating O-GlcNAc in signaling and other fundamental biological pathways. J Biol Chem.

[23] Ji W (2024). Metabolomic approaches to dissect dysregulated metabolism in the progression of pre-diabetes to T2DM. Mol Omics.

[24] Marshall S (1991). Discovery of a metabolic pathway mediating glucose-induced desensitization of the glucose transport system. Role of hexosamine biosynthesis in the induction of insulin resistance. J Biol Chem.

[25] Oguchi M (1975). Phosphorylation of D-glucosamine by rat liver glucokinase. J Biochem.

[26] Torres CR, Hart GW (1984). Topography and polypeptide distribution of terminal N-acetylglucosamine residues on the surfaces of intact lymphocytes. Evidence for O-linked GlcNAc. J Biol Chem.

[27] Haltiwanger RS (1990). Enzymatic addition of O-GlcNAc to nuclear and cytoplasmic proteins. Identification of a uridine diphospho-N-acetylglucosamine:peptide beta-N-acetylglucosaminyltransferase. J Biol Chem.

[28] Wu C (2024). OGT and OGA: sweet guardians of the genome. J Biol Chem.

[29] Alejandro EU (2015). Disruption of O-linked N-acetylglucosamine signaling induces ER stress and β cell failure. Cell Rep.

[30] Mohan R (2021). OGT regulates mitochondrial biogenesis and function via diabetes susceptibility gene Pdx1. Diabetes.

[31] Chong CM (2022). Presenilin-1 F105C mutation leads to tau accumulation in human neurons via the Akt/mTORC1 signaling pathway. Cell Biosci.

[32] Silva-Aguiar RP (2022). O-GlcNAcylation in renal (patho)physiology. Int J Mol Sci.

[33] Packer M (2023). Fetal reprogramming of nutrient surplus signaling, O-GlcNAcylation, and the evolution of CKD. J Am Soc Nephrol.

[34] Jo S (2024). Loss of O-GlcNAcylation modulates mTORC1 and autophagy in β cells, driving diabetes 2 progression. JCI Insight.

[35] Eisenhardt AE (2016). Phospho-proteomic analyses of B-Raf protein complexes reveal new regulatory principles. Oncotarget.

[36] Carracedo A (2008). Inhibition of mTORC1 leads to MAPK pathway activation through a PI3K-dependent feedback loop in human cancer. J Clin Invest.

[37] Zhang S (2021). AKAP13 couples GPCR signaling to mTORC1 inhibition. PLoS Genet.

[38] Hasegawa J (2022). PIKFYVE-dependent regulation of MTORC1 and TFEB. Autophagy Rep.

[39] Hill EV (2010). Regulation of PIKfyve phosphorylation by insulin and osmotic stress. Biochem Biophys Res Commun.

[40] Bagaria J (2022). Genetics, functions, and clinical impact of presenilin-1 (PSEN1) gene. Int J Mol Sci.

[41] Harris TE (2006). mTOR-dependent stimulation of the association of eIF4G and eIF3 by insulin. EMBO J.

[42] Avruch J (2006). Insulin and amino-acid regulation of mTOR signaling and kinase activity through the Rheb GTPase. Oncogene.

[43] Petit CS (2013). Recruitment of folliculin to lysosomes supports the amino acid-dependent activation of Rag GTPases. J Cell Biol.

[44] Tsun ZY (2013). The folliculin tumor suppressor is a GAP for the RagC/D GTPases that signal amino acid levels to mTORC1. Mol Cell.

[45] Kim S (2021). Leucine-sensing mechanism of leucyl-tRNA synthetase 1 for mTORC1 activation. Cell Rep.

[46] Rogacka D (2010). Expression of GFAT1 and OGT in podocytes: transport of glucosamine and the implications for glucose uptake into these cells. J Cell Physiol.

[47] Oslowski CM (2012). Thioredoxin-interacting protein mediates ER stress-induced β cell death through initiation of the inflammasome. Cell Metab.

[48] Giri PR (1992). Molecular and phylogenetic analysis of calmodulin-dependent protein phosphatase (calcineurin) catalytic subunit genes. DNA Cell Biol.

[49] Heit JJ (2006). Calcineurin/NFAT signalling regulates pancreatic beta-cell growth and function. Nature.

[50] Taneera J (2024). Unraveling the significance of PPP1R1A gene in pancreatic β-cell function: a study in INS-1 cells and human pancreatic islets. Life Sci.

[51] Taneera J (2026). Impact of PPP1R1A knockdown on the proteomic landscape of INS-1 cells: a focus on significant modulated Pathways. J Proteome Res.

[52] Zhang CS (2017). Fructose-1,6-bisphosphate and aldolase mediate glucose sensing by AMPK. Nature.

[53] Zhang CS (2014). The lysosomal v-ATPase-Ragulator complex is a common activator for AMPK and mTORC1, acting as a switch between catabolism and anabolism. Cell Metab.

[54] Li M et al (2021). Aldolase is a sensor for both low and high glucose, linking to AMPK and mTORC1. Cell Res.

[55] Almacellas E (2019). Phosphofructokinases axis controls glucose-dependent mTORC1 activation driven by E2F1. iScience.

[56] Orozco JM (2020). Dihydroxyacetone phosphate signals glucose availability to mTORC1. Nat Metab.

[57] Lee MN (2009). Glycolytic flux signals to mTOR through glyceraldehyde-3-phosphate dehydrogenase-mediated regulation of Rheb. Mol Cell Biol.

[58] Liu X (2024). Regulation of protein O-GlcNAcylation by circadian, metabolic, and cellular signals. J Biol Chem.

[59] Kim K (2022). O-GlcNAc modification of leucyl-tRNA synthetase 1 integrates leucine and glucose availability to regulate mTORC1 and the metabolic fate of leucine. Nat Commun.

[60] Durgan DJ (2011). O-GlcNAcylation, novel post-translational modification linking myocardial metabolism and cardiomyocyte circadian clock. J Biol Chem.

[61] Liu X (2021). Hexosamine biosynthetic pathway and O-GlcNAc-processing enzymes regulate daily rhythms in protein O-GlcNAcylation. Nat Commun.

[62] Balboa D (2018). Insulin mutations impair beta-cell development in a patient-derived iPSC model of neonatal diabetes. Elife.

[63] Weimer S (2014). D-Glucosamine supplementation extends life span of nematodes and of ageing mice. Nat Commun.

[64] Li F (2023). Glucosamine improves non-alcoholic fatty liver disease induced by high-fat and high-sugar diet through regulating intestinal barrier function, liver inflammation, and lipid metabolism. Molecules.

[65] Shintani H (2018). Calorie restriction mimetics: upstream-type compounds for modulating glucose metabolism. Nutrients.

[66] Pasternak CA (1991). Regulation of glucose uptake by stressed cells. J Cell Physiol.

[67] Lan R (2016). Mitochondrial pathology and glycolytic shift during proximal tubule atrophy after ischemic AKI. J Am Soc Nephrol.

[68] Suh HN (2014). Glucosamine-induced Sp1 O-GlcNAcylation ameliorates hypoxia-induced SGLT dysfunction in primary cul-tured renal proximal tubule cells. J Cell Physiol.

[69] Tran DH (2020). Chronic activation of hexosamine biosynthesis in the heart triggers pathological cardiac remodeling. Nat Commun.

[70] Heerspink HJL (2020). Dapagliflozin in patients with chronic kidney disease. N Engl J Med.

[71] Perkovic V (2019). Canagliflozin and renal outcomes in type 2 diabetes and nephropathy. N Engl J Med.

[72] Zinman B (2015). Empagliflozin, cardiovascular outcomes, and mortality in type 2 diabetes. N Engl J Med.

