# Supplementary material for: Glucosamine links hyperglycemia to mTORC1 activation and glucose toxicity in diabetes
Source: JCI Insight. 2026 May 22;11(10):e197331. doi: 10.1172/jci.insight.197331 (PMC13232723; doi:10.1172/jci.insight.197331)
Supplement: Supplemental data [file jciinsight-11-197331-s100.pdf]

Supplemental data

Extended Methods

Metabolic tests

IPGTT was performed following an overnight fast by i.p. injection of glucose (1.3 g/kg), followed by consecutive measurements of tail blood glucose at the indicated time points. Blood samples for plasma insulin measurements were collected from the facial vein. Insulin levels were analysed using ultrasensitive ELISA kits (Crystal Chem, USA).

LC-MS metabolomics analysis

For the measurements of metabolites in the kidney, liver, heart, and plasma, mice were fasted for 1 hour before receiving three consecutive i.p. injections of $^{13}C_6$-D-glucose (1 mg/g) (Sigma-Aldrich) at 0, 20, and 30 min. Mice were then sacrificed by cervical dislocation 10 min after the final injection. Tissues were collected immediately, snap frozen in liquid nitrogen, and stored at -80°C. To analyze plasma metabolites, approximately 30 µL of blood was drawn from the tail into heparin-coated tubes just before the mice were sacrificed. The plasma was separated, snap-frozen in liquid nitrogen, and stored at -80°C.

Frozen kidney cortex, livers, and hearts weighing ~30 mg were transferred into soft tissue homogenizing CK 14 tubes containing 1.4 mm ceramic beads (Bertin Corp.) prefilled with 300 µl of cold (-20°C) metabolite extraction solvent (methanol:acetonitrile:water, 5:3:2, respectively) and kept on ice. Samples were homogenized using Precellys 24 tissue homogenizer (3 cycles of 20 sec at 6,000 rpm, with a 30-sec gap between each of cycles; Bertin Technologies) cooled to 4°C. Homogenized extracts were centrifuged in the Precellys tubes at 18,000g for 15 min at 4°C; supernatants were collected in microcentrifuge tubes and centrifuged again at 18,000g for 10 min at 4°C. The supernatants were transferred to glass high performance liquid chromatography (HPLC) vials and kept at -75°C prior to LC-MS analysis. Plasma was diluted in a ratio of 1:10 with cold (-20°C) metabolite extraction solvent (methanol/acetonitrile, 75:25) and vortexed for 10 min. Samples were then treated

as described above for tissue homogenized extracts and kept at -75°C prior to LC-MS analysis.

For metabolite measurements in primary KPTCs and MIN6, cells were cultured in complete DMEM medium supplemented with 5 or 25 mM glucose, GlcN 5mM, rapamycin 10nM or azaserine for 48h. Following, cells were washed twice with PBS and incubated in DMEM with 5 mM or 25mM $^{13}C_6$-labelled D-glucose (Sigma-Aldrich; Cat# 389374) for 3h or 4mM L-Glutamine-$^{13}C_5$,$^{15}N_2$ (Sigma-Aldrich; Cat# 607983) for 6h. The plates were washed twice with ice-cold PBS, and 900 µl of cold metabolite extraction solvent (methanol:acetonitrile:water, 5:3:2, respectively) was added. The plates were rotated for 15 min at 4 °C. The cell extracts were collected into microcentrifuge tubes and recentrifuged at 18,000 x g for 10 min. The supernatants were transferred to glass HPLC vials and stored at -80 °C prior to LC-MS analysis. Protein concentrations were determined using the Pierce™ BCA Protein Assay Kit (Thermo Scientific, IL, USA).

For metabolite measurements in human islets, the islets were cultured for 48 h in PIM growth medium containing either 5.8mM (low glucose, LG) or 25mM glucose (high glucose, HG), or in LG medium supplemented with varying concentrations of glucosamine. The islets were then washed with serum-free and glucose -free RPMI medium containing Hepes, 0.02% BSA, and sodium pyruvate, followed by incubation in the same medium with either LG or HG of ^{13}C-glucose for 2.5 h, according to the long-term incubation protocol. After incubation, the islets were washed twice with ice-cold PBS, and 150 µl of cold metabolite extraction solvent (methanol:acetonitrile, 5:3:2, respectively) was added. The tubes were rotated for 15 min at 4°C, and cell extracts were centrifuged at 14,000 rpm for 10 min. The supernatants were transferred to glass HPLC vials and stored at -80°C until LC-MS analysis. Protein concentrations were determined using the Pierce™ BCA Protein Assay Kit (Thermo Scientific, IL, USA).

LC-MS metabolomics analysis for polar metabolites profiling was performed as described previously (1). Briefly, the Thermo Vanquish Flex ultra-high-performance liquid chromatography (UPLC) system coupled to Orbitrap Exploris 240 Mass Spectrometer (Thermo Fisher Scientific) was used. Resolution was set to 120,000 at 200 m/z with electrospray ionization and polarity switching mode to enable both positive and negative ions across a mass range of 67-1,000 m/z. UPLC setup consisted of ZIC-pHILIC column (Merck). Biological extracts (5 µl) were injected, and the compounds were separated using a mobile phase gradient of 15 min, starting at 20% aqueous (20 mM ammonium carbonate adjusted to pH 9.2 with 0.1% of 25% ammonium hydroxide)/80% organic (acetonitrile) and terminated with 20% acetonitrile. Flow rate and column temperature were maintained at 0.2 ml/min and 45°C, respectively, for a total run time of 27 min. For glucose and glucosamine detection, a different analytical method was used. Thermo Vanquish Flex ultra-high-performance liquid chromatography (UPLC) system coupled to IQ-X Mass Spectrometer (Thermo Fisher Scientific) was used. Resolution was set to 90,000 at 200 m/z with electrospray ionization and polarity switching mode to enable both positive and negative ions across a mass range of 67-1,000 m/z. UPLC setup consisted of BEH-Amide column (Waters). Biological extracts (5 µl) were injected, and the compounds were separated using isocratic conditions for 15 minutes at 15% aqueous (0.1% formic acid)/85% organic (acetonitrile) followed by wash and equilibration. Flow rate and column temperature were maintained at 0.2 ml/min and 40°C, respectively, for a total run time of 25 min. For quantifiying glucose and glucosamine, labeled analogues ($^{13}C_6$-Glucose, $^{13}C_6$-Glucosamine) were spiked into the media and intracellular matrix at known concentration to create a calibration curve with the proper matrix effect. Concentrations were calculated based on linear equation. All metabolites were detected using mass accuracy below 1 ppm. Thermo Xcalibur 4.4 was used for data acquisition. TraceFinder 4.1 was used for data analysis. Peak areas of metabolites were determined by using the exact mass of the singly charged ions. The peak areas of different metabolites were determined using Thermo TraceFinder™ 4.1 software, where metabolites were identified by the exact mass of the singly charged ion and by known retention time,

using an in-house MS library built by running commercial standards of all detected metabolites. For data normalization, raw data files were processed with Compound Discoverer 3.1 to obtain total measurable ion peak intensities for each sample. Each identified metabolite intensity was normalized to the total intensity of the sample. Metabolite-Auto Plotter v2.6 was used for data visualization during data processing.

KPTC and HK-2 cell cultures

Primary mouse KPTCs used in this study were isolated using the following protocol: Mouse kidney cortices were dissociated into single cells using 0.7 mg/ml collagenase/dispase (Sigma-Aldrich; Cat# 10269638001) in Hanks' Balanced Salt Solution (HBSS) followed by gentle vortex. Red blood cells were removed using RBC Lysis solution (Sartorius; Cat #01-888-1B). KPTCs were then purified through low-speed centrifugation (100 x g) and cultured in a REGM BulletKit medium (Lonza; Cat #CC-3191 & #CC-4127) on collagen-coated plates (2). HK-2 cells were cultured in DMEM supplemented with 4.5g/l glucose with 10% fetal bovine serum (FBS) and 100 IU/ml penicillin/streptomycin (Pen/Strep. Sartorius) at 37°C in a humid atmosphere with 5% CO_2.

Islet isolation, β-cell line culture, and experimental protocols

The cell line MIN6 derived from insulinoma in transgenic mice expressing SV40 T antigen under the insulin promoter and originally established by Prof. Jun-Ichi Miyazaki (Osaka University, Japan). Cells were expanded in DMEM supplemented with 4.5g/l glucose, 15% heat-inactivated FBS, 100 IU/ml Pen/Strep. solution, 2 mM 1-glutamine, 1 mM sodium pyruvate (Biological Industries, Israel) and 0.05 mM 2-mercaptoethanol (Sigma-Aldrich) in a humidified 37°C, 5% CO_2 incubator. Mycoplasma contamination was examined

periodically and the tests showed no evidence for contamination. Functionality of the cell line was validated by checking periodically their glucose stimulated insulin secretion.

Mice islets were isolated by collagenase P (Roche Diagnostics, Germany) injection into the pancreatic duct followed by Histopaque (Sigma-Aldrich, USA) density gradient separation. Islets were hand-picked and unless mentioned cultured overnight in complete RPMI-1640 medium containing 11 mM glucose supplemented with 10% heat-inactivated FBS, 100 IU/ml Pen/Strep. solution, 10 mM HEPES, 2 mM 1-glutamine, 1 mM sodium pyruvate and 0.05 mM 2-mercaptoethanol in a humidified 37°C, 5% CO_2 incubator.

Islets from donors were obtained from Prodo Laboratories (details on donor and islet characteristics are provided in Supplemental Table 1) and cultured in PIM(S) Standard Islet Media (Prodo Laboratories) supplemented with 5% human albumin serum at a glucose concentration of 5.8 mM prior to the metabolomic experiment.

For static glucose-stimulated insulin secretion tests, batches of 15-25 islets in 4 or 5 replicates were pre-incubated for 60 min in 100 µl RPMI-1640 containing 3.3 mM glucose, then consecutively incubated at 3.3 mM and 16.7 mM glucose for 1 hr at 37°C in 100 µl modified Krebs-Ringer bicarbonate buffer containing 20 mM HEPES and 0.25% BSA (KRBH-BSA). Medium was collected, centrifuged, and frozen at -20°C and islets were lysed using 50 µl 0.1% BSA-GB/NP-40. Insulin in medium and islet lysates was determined by ELISA.

Seahorse analysis

The oxygen consumption rate (OCR) and the extracellular acidification rate (ECAR) of primary KPTCs, as well as the OCR of dispersed mouse islets, were determined using an Agilent Seahorse XFe96 Analyzer and the XF cell mito stress test kit (Agilent Technologies, USA). KPTCs were cultivated in 96-well collagen-coated assay plates at a density of 40 x

10^3 cells/well in DMEM growth medium (2% FBS; 1% glutamine; 1% P/S; 1% pyruvate; 5 or 25 mM glucose) for 48 h.

Mouse islets, dispersed with accutase solution (Sartorius), were seeded on 96-well poly-lysine coated assay plates at a density of 40 islets/well in RPMI growth medium (11 mM glucose, 10% FBS; 1% glutamine; 1% P/S; 1% pyruvate with beta mercapto ethanol) and were allowed to adhere overnight in a 37°C humidified atmosphere of 5% CO_2.

Cell-free wells were treated with the same medium for background correction. The following day, the medium was replaced with DMEM (for KPTCs) or RPMI (for islets) treatment mediums with specified glucose concentrations. When indicated, GlcN and rapamycin were added at the stated concentrations for 48 h. Before starting the assay, the growth medium was changed to unbuffered serum-free DMEM, pH 7.4 (Agilent Seahorse XF; Cat# 103575-100) for KPTCs or serum-free RPMI pH 7.4 (Agilent Seahorse XF; Cat # 103576-100) for dispersed islets with the same medium composition, and the cells were incubated at 37°C without CO_2 for 1 hour to equilibrate. OCR and ECAR were measured simultaneously in repeated cycles to obtain basal rates. Following baseline measurements, OCR was assessed following the injection of oligomycin (1.5 μM). Subsequently, carbonylcyanide-4-(trifluoromethoxy) phenylhydrazone (FCCP, 1 μM) was injected, and the maximal OCR was recorded. Non-mitochondrial oxygen consumption was measured after injecting rotenone and antimycin A (0.5 μM each). For KPTCs, specific timepoints were 3 min mixing and 3 min of measurements. To prevent detachment of dispersed islets, the timepoints were adjusted to 1 min mixing, 2 min waiting, and 3 min of measurements. OCR and ECAR rates were normalized to the total cell protein concentration, which was quantified using the Pierce™ BCA Protein Assay Kit (Thermo Scientific, IL, USA).

Western blotting

Kidney cortex or cell homogenates were prepared in RIPA buffer supplemented with protease and phosphatase inhibitor cocktails (sigma aldrich) and Phenylmethanesulfonyl Fluoride (PMSF, 1mM), followed by protein concentration measurement using the Bradford assay (Bio-Rad). Samples were resolved by 10% or 8% SDS-PAGE and transferred to nitrocellulose membranes. After blocking for 1 h in 5% milk, blots were incubated overnight with rabbit anti-pS6 (5364S, Cell Signaling Technology), S6 (2217S, Cell Signaling Technology), 4E-BP1 (9644, Cell Signaling Technology), p4E-BP1 (9459, Cell Signaling Technology), pAMPK (Thr172; 2535, Cell Signaling Technology), AMPK (2532, Cell Signaling Technology), pULK1 (6888, Cell Signaling Technology), ULK (8054, Cell Signaling Technology), a-tubulin (2144, Cell Signaling Technology), pAKT(Ser473; 4060 Cell signaling Technology AKT (9272, Cell signaling Technology) and mouse anti-GAPDH (ab8245, Abcam) antibodies at 4°C. Anti-rabbit/mouse horseradish peroxidase (HRP)-conjugated secondary antibodies (Jackson ImmunoResearch) were used for 1 h at room temperature, followed by chemiluminescence detection with Clarity Western ECL Blotting Substrate (Bio-Rad, CA) using the Chemidoc Touch Imaging System. Relative band intensities were quantified by densitometric analysis using the ImageJ software (NIH) and image Lab Software version 6.1 (Bio-Rad Laboratories).

Immunofluorescence staining of pancreatic sections and cells

Pancreases were fixed with 4% formaldehyde overnight. Paraffin-embedded sections were deparaffinised and rehydrated, and antigen retrieval was performed in citrate buffer (10 mM citric acid, Bio-Lab; 41.5 mM sodium citrate dihydrate, Avantor MACRON, USA; pH 6). Sections were blocked with CAS-Block for 10 min (Thermo Fisher Scientific, USA). The following primary antibodies diluted in CAS-Block were used for immunostaining: guinea pig anti-insulin 1:5 (IR0026, Agilent Technologies, USA), mouse anti-glucagon 1:200 (ab10988, Abcam, UK**),** rabbit anti-phosphorylated S6 ribosomal protein (pS6) 1:200 (5364, Cell Signaling Technology, USA), rabbit anti-PDX-1 1:200 (5679, Cell Signaling Technology),

rabbit anti-NKX6.1 1:100 (54551, Cell Signaling Technology), rabbit anti-BIP 1:200 (3177, Cell Signaling Technology). Secondary antibodies including Cy2- conjugated anti guinea pig, Cy5-conjugated anti rabbit anti Cy3-conjugated anti mouse were all from Jackson ImmunoResearch (USA) and were diluted 1:200 in 1% (wt/vol.) BSA in PBS. Cell nuclei were visualized with DAPI staining. Digital images were obtained with a Nikon A1R confocal microscope (magnification of x40). The image files were processed using QuPath software 0.4.3.

To determine β-cell area, consecutive paraffin sections 75 μm apart, spanning the entire pancreas, were analyzed; slides were scanned using the Zeiss Axio Scan.Z1 slide scanner through a x20 objective using identical imaging conditions. The image files were processed using QuPath software 0.4.3 and the percent area covered by insulin was determined.

For staining of MIN6 and KPTCs 5 x 10^4 cells were plated on each polylysine or collagen coated glass coverslip the day before the experiment. Cells were fixed with 4% paraformaldehyde in PBS for 15 min, washed with PBS three times and permeabilized with 0.1% Triton X-100 in PBS for 10 min. Cells were washed once with PBS and blocked with 5% BSA in PBS for 30 min at room temperature. The coverslips were then incubated with primary antibodies against mTOR, LAMP2 or pS6 overnight at 4°C. The coverslip was rinsed three times and incubated with secondary antibodies for 2 h at room temperature. For pS6 staining, coverslips were washed and mounted using DAPI Fluoromount-G (0100-20, SouthernBiotech). Images were acquired using a Nikon A1R confocal microscope at 40x magnification, and fluorescence intensity was quantified using ImageJ (NIH).

For mTORC1-LAMP2 colocalization analysis, coverslips were mounted with Prolong Glass Antifade Mountant with NucBlue (ThermoFisher). High-resolution images were obtained with an Airyscan confocal microscope. Colocalization was quantified using

QuPath version 0.5.1 by measuring the intersection area of mTORC1-LAMP2 positive signals.

Mitochondrial Labeling with MitoTracker Red CMXRos in MIN6 Cells

MIN6 cells were seeded on collagen-coated glass coverslips. The following day, cells were treated for 24 hours with or without 1 mM glucosamine and 20 µM MitoTempo in DMEM supplemented with 2% FBS, 1% glutamine, 1% penicillin/streptomycin, 1% pyruvate, and 3 mM glucose (13-mercaptoethanol omitted). Cells were then incubated with 800 nM MitoTracker Red CMXRos (Invitrogen, ThermoFisher Scientific) for 45 minutes, according to manufacturer's instructions. After staining, the medium was replaced with the respective treatment medium for 30 minutes. Cells were washed with PBS, fixed in 4% PFA for 20 minutes, and washed again with PBS. MitoTracker probe fluorescence intensity was analyzed using a Nikon A1R confocal microscope.

Flow cytometry

Islets from 6-week-old Akita mice, treated or untreated with dapagliflozin for 2 weeks (100 islets per group), were first incubated in RPMI medium with 1mM glucose, 1% heat-inactivated FBS, and 10 mM HEPES for 30 min, then in the same medium with 16.7 mM glucose for 1 hour. Islets were dissociated into a single-cell suspension using Accutase; subsequently, cells were fixed with Cytofix/Cytoperm solution (8D Biosciences, USA) for 20 min, washed twice with Perm/Wash buffer (8D Biosciences), and incubated overnight at 4°C with guinea pig anti-insulin antibody (A0564, Agilent Technologies) and rabbit anti-pS6 antibody (#5364, Cell Signaling Technology). The cells were then washed twice, incubated for 2 h with species-specific secondary antibodies conjugated to Cy2 and Cy5, respectively (Jackson ImmunoResearch), washed two more times, and resuspended in 250 µl PBS. They were filtered through a 40 mm nylon mesh into flow cytometry tubes and analysed using an LSR-Fortessa flow cytometer (8D Biosciences), recording at least 10,000 events.

Quantification was performed using FlowJo software v10.8.0 (https://www.flowjo.com/solutions/flowjo/downloads). Results are presented as the percentage of pS6+ beta cells and mean fluorescence intensity of pS6 in β-cells.

RNA extraction and quantitative real-time PCR

Total RNA was extracted from MIN6 cells using TRI Reagent (Biolab), and from mouse islets using QIAshredders and the RNeasy Micro Kit (Qiagen), following the manufacturers' protocols. cDNA was synthesized from RNA using the qScript High-Capacity cDNA Reverse Transcription Kit (Quantabio, Beverly, MA). Quantitative real-time PCR (qRT-PCR) was performed using FastStart SYBR Green Master Mix (Applied Biosystems, USA) on a QuantStudio 5 Real-Time PCR System (Applied Biosystems, USA). Gene expression levels were normalized to *Actb* expression. Primer sequences used for qPCR are provided in Supplemental Table 1.

siRNA-Mediated Knockdown of *Gfat1*

For the knockdown of *Gfat1,* MIN6 cells were transfected with an ON-TARGETplus SMARTpool siRNA targeting *Gfat1* or an ON-TARGETplus Non-targeting control pool siRNA (Dharmacon). Cells were seeded in 6-well plates and grown to 80% confluency. Transfection was performed using the *Trans*IT-X2™ Dynamic Delivery System (Mirus). Briefly, cells were transfected with 25 nM of either *Gfat1* siRNA or non-targeting control siRNA using 7.5 μl of the transfection reagent per well, according to the manufacturer's protocol. After 48 hours of incubation, the cells were harvested for RT-PCR to quantify *Gfat1* mRNA levels, Western blotting to analyze the mTORC1 signaling pathway and metabolomic analysis of downstream metabolites in the hexosamine pathway. The sequences for the *Gfat1* siRNA pool, the non-targeting control pool, and the primers used for RT-PCR are listed in Supplemental Table 1.

RNA-seq data processing and analysis

Pancreatic islets were isolated from three WT mice, using a split-sample design; islets from each mouse were divided equally into two groups, receiving either 0.5 mM Glucosamine or vehicle control for 24 hours. This yields three paired biological replicates per condition. Following treatment, islets were dissociated into single-cell suspension using Accutase. Nuclei were subsequently isolated according to the 10x Genomics Demonstrated Protocol ("Nuclei Isolation for Single Cell Multiome ATAC + Gene Expression Sequencing", CG000365"). During the lysis buffer incubation, manual dissociation was performed by passing the suspension through a 30G needle with a 1-ml syringe to facilitate nuclear release and isolation. The scRNA libraries were prepared using the Chromium Next GEM Single Cell Multiome ATAC and Gene expression reagent Kit (10x Genomics). Nuclei were diluted in 5 ul and proceeded to the transposition mix and incubated for one hour at 37°C and according to the company manufacture (10x Genomics). Then the mix was separated into droplet emulsion using a Chromium X machine (10x Genomics) and libraries were prepared according to the manufacturer's protocol. The scRNA-seq libraries were sequenced on the Illumina Novaseq6000 sequencing system (Illumina) as a 28-bp and 90-bp paired-ended.

Analysis of the snRNA-seq transcriptome was performed using the Seurat v5 package within the R environment using RStudio (https://cran.r-project.org/src/base/R-4/R-4.5.0.tar.gz). Quality control filters were applied to ensure data integrity: specifically, nuclei were retained with feature counts >500 and a mitochondrial gene content <5%. To minimize the Impact of inter-individual variability, correction by mouse origin was applied using the VarsToRegress option. Following normalization and scaling, cell populations were clustered using Seurat's shared nearest neighbor (SNN) algorithm. The β-cell cluster was identified and validated based on the expression of canonical markers, including *Ins1* and *Ins2.* Differentially expressed genes (DEGs) between glucosamine-treated or untreated control β-cell nuclei were identified within the β-cell population using a minimum fraction (min.pct) of 0.3 and a log2FC 0.25. Functional enrichment of these DEGs was further characterized by Gene Ontology (GO) analysis.

RNA intended for RNA-seq of WT, Akita and Akita islets treated with dapagliflozin was extracted on the day of islet isolation. RNA was sequenced using Novaseq6000 (Run details 122bp single end read). Raw sequencing reads were obtained from the NextSeq system and converted to FASTQ files using bcl2fastq (v2.20.0.422) with default parameters. Quality control of the raw reads was assessed using FastQC (v0.11.8). Raw reads were processed for quality trimming and adaptors removal using fastx_toolkit v0.0.14 and cutadapt v2.10. The processed reads were aligned to the mouse transcriptome and genome version GRCm39 with annotations from Ensembl release 106 using TopHat v2.1.1 (3). Counts per gene quantification was done with htseq-count v2.01 (4). Normalization and differential expression analysis were done with the DESeq2 package v 1.36.0 (5). Pair-wise comparisons were tested with default parameters (Wald test), without applying the independent filtering algorithm. Significance threshold was taken as padj<0.1. In addition, significant DE genes were further filtered by the log2FoldChange value. This filtering was baseMean-dependent and required a baseMean above 5 and an absolute log2FoldChange higher than 5/sqrt(baseMean) + 0.3 (for highly expressed genes this means a requirement for a fold-change of at least 1.2, while genes with a very low expression would need a 5.8 -fold change to pass the filtering).

Phosphoproteomics

MIN6 cells were incubated for 2h in starvation medium then stimulated for 30 min with medium including 1mM glucosamine. Cells were then washed three times in cold PBS then lysed directly on the plate with 5% SDS, 50mM Tris pH 7.6 buffer. Protein concentration was determined using BCA kit. 100µg protein were taken for phosphoenrichment.

The samples were then subjected to tryptic digestion using an S-trap and enriched on IMAC beads using a Bravo automated platform. The resulting peptides were analyzed using nanoflow liquid chromatography (NanoElute2) coupled to high resolution, high mass

accuracy mass spectrometry (TIMS-Tof Pro) in high sensitivity mode. Each sample was analyzed on the instrument separately in a random order in DDA-PASEF mode.

Raw data were processed with FragPipe v23.1. The data were analyzed against the murine Uniprot database appended with common lab protein contaminants in LFQ-MBR workflow and with the default modification settings with the addition of pSTY. The phosphopeptides intensities were calculated and used for generating the phospho sites intensities with an in-house script. The calculated intensities were used for further analysis in Perseus v1.6.2.3. They were log2 transformed and only sites that had at least 2 valid values in at least one experimental group were kept. The remaining missing values were imputed using a low constant value. A student's t-test was first performed to identify sites differentially phosphorylated by glucosamine compared to control. We then assessed how these glucosamine-responsive sites were modulated by the addition of the OGTi to identify OGT-dependent phosphorylation events.

References

1. Mackay GM, Zheng L, van den Broek NJ, and Gottlieb E. Analysis of Cell Metabolism Using LC-MS and Isotope Tracers. *Methods Enzymol.* 2015;561:171–96.
2. Permyakova A, Hamad S, Hinden L, Baraghithy S, Kogot-Levin A, Yosef O, et al. Renal Mitochondrial ATP Transporter Ablation Ameliorates Obesity-Induced CKD. *J Am Soc Nephrol.* 2024;35(3):281–98.
3. Kim D, Pertea G, Trapnell C, Pimentel H, Kelley R, and Salzberg SL. TopHat2: accurate alignment of transcriptomes in the presence of insertions, deletions and gene fusions. *Genome Biol.* 2013;14(4):R36.
4. Anders S, Pyl PT, and Huber W. HTSeq--a Python framework to work with high-throughput sequencing data. *Bioinformatics.* 2015;31(2):166–9.
5. Love MI, Huber W, and Anders S. Moderated estimation of fold change and dispersion for RNA-seq data with DESeq2. *Genome Biol.* 2014;15(12):550.

Supplemental data

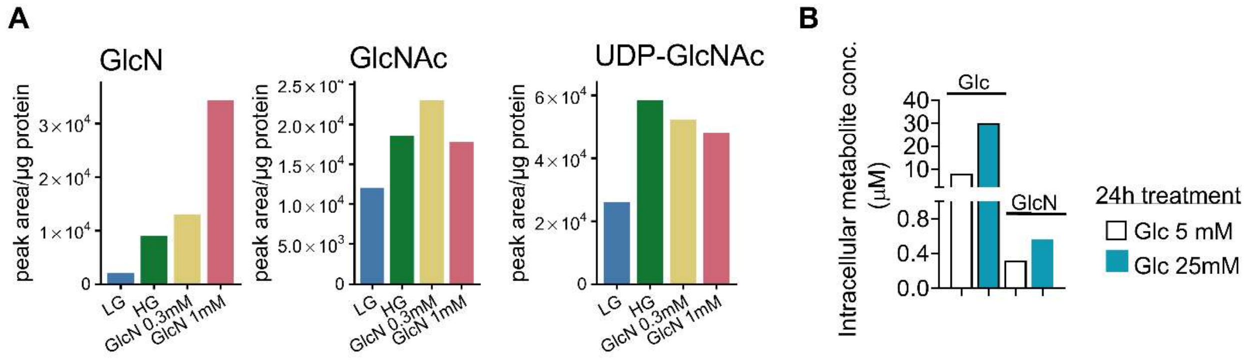

Supplemental Figure 1. (**A**) Levels of GlcNAc and hexosamine pathway metabolites in human islets cultured at LG, HG or LG with glucosamine (0.3 or 1mM) for 48h. (**B**) Intracellular concentration of glucose and glucosamine in human islets treated with 5 or 25mM glucose for 24h. Glucose and glucosamine levels were quantified using a calibration curve generated from standards of known concentrations.

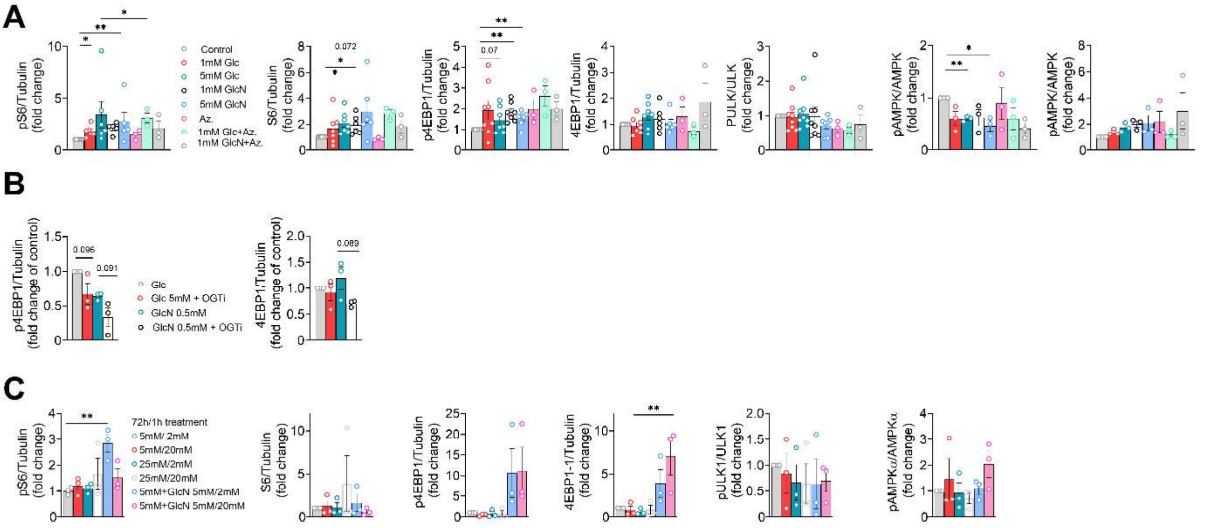

Supplemental Figure 2. Quantification of protein levels from the Western blots shown in (**A**) Fig. 3C, (**B**) Fig. 3E, and (**C**) Fig. 3G. Data are presented as mean ± SEM and analyzed by 1-way ANOVA or unpaired t-test. *P < 0.05, **P < 0.01.

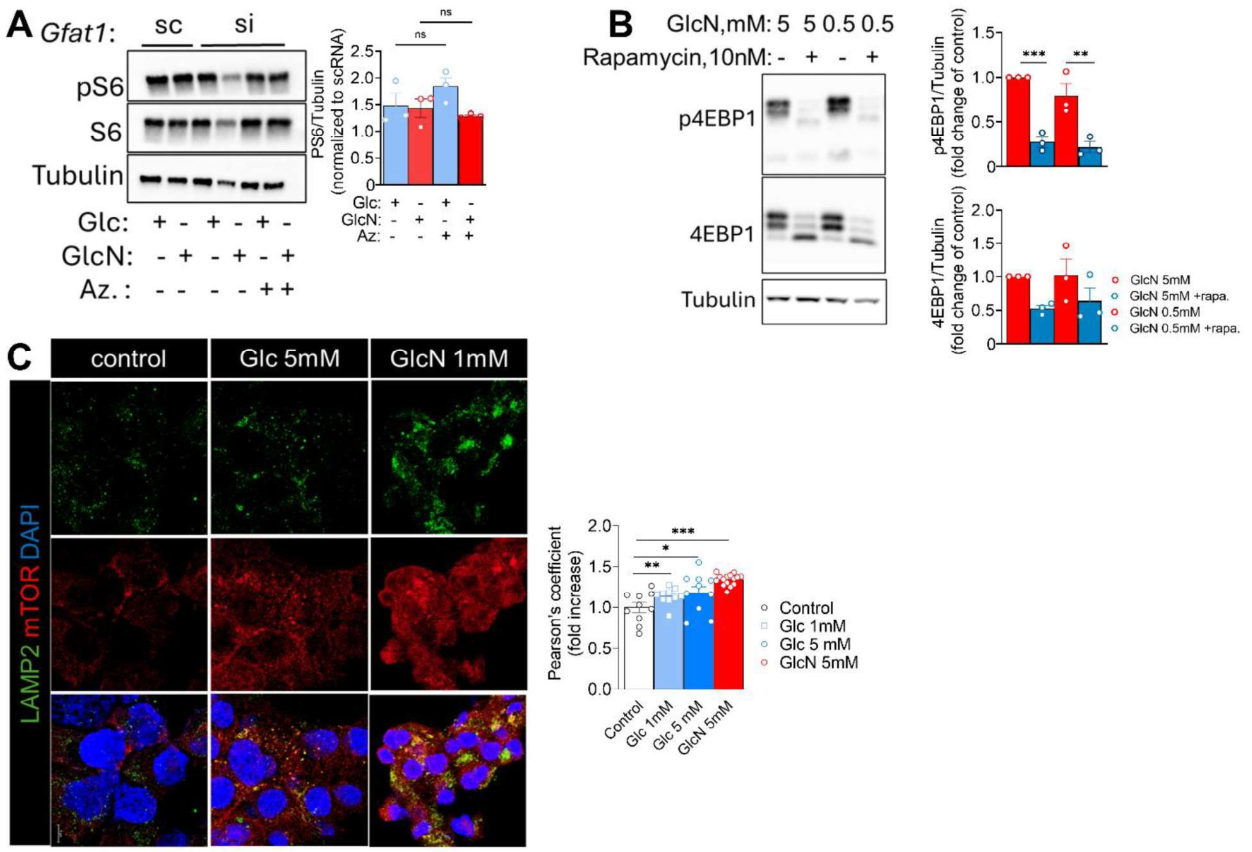

Supplemental Figure 3. (A) MIN6 cells were transfected with either scrambled or *Gfat1*-targeting siRNA and then treated as described in Fig. 2D. mTORC1 activity was assessed by Western blotting for phosphorylated and total S6 and tubulin (housekeeping). (**B**) MIN6 cells were treated with the indicated concentrations of glucosamine with or without 10 nM rapamycin for 48 h followed by Western blotting for total and phosphorylated 4EBP1 and tubulin. (**C**) MIN6 cells were incubated in starvation medium for 2 h and then with glucose or glucosamine at the indicated concentrations. Immunofluorescence for mTOR and LAMP2. The percentage of mTORC1$^+$ signal overlapping with the LAMP2$^+$ area, relative to the total LAMP2$^+$ area, is shown. Microscopic analysis was performed on 9 to 11 fields using the Airyscan system. Data represent the mean ± SEM. Data were analyzed by 1-way ANOVA. *P < 0.05, **P < 0.01, ***P < 0.001.

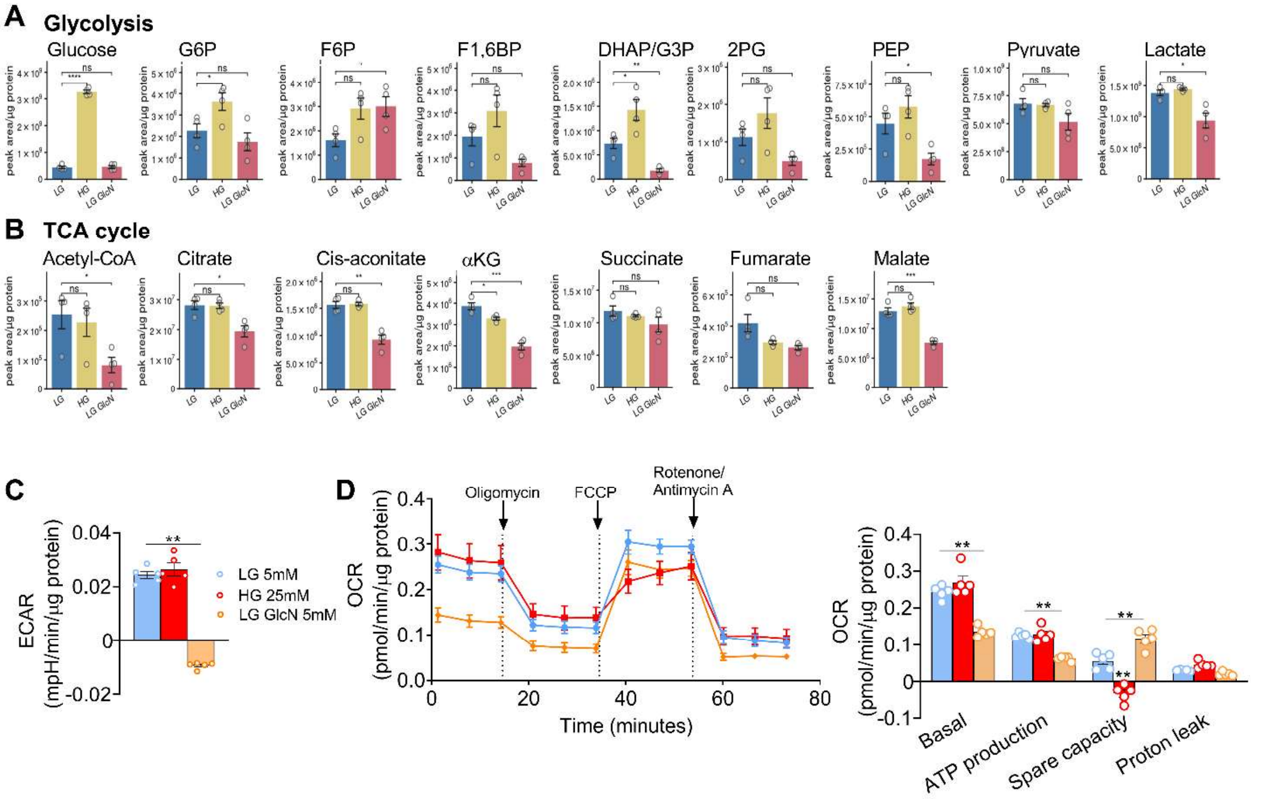

Supplemental Figure 4. Primary KPTCs were cultured under the following conditions: LG, HG or LG with glucosamine (5mM) for 48h. (**A**) Relative abundance of glucose and glycolytic intermediates. (**B**) Relative abundance of tricarboxylic acid (TCA) cycle metabolites. (**C**) Extracellular acidification rate (ECAR) and (**D**) oxygen consumption rate (OCR) measured by the Seahorse XF system. OCRs were measured at baseline, following 1.5μM oligomycin, 1μM FCCP, and 0.5μM antimycin/rotenone. (n=3). Data represent the mean ± SEM. Data were analyzed by 1 and 2-way ANOVA. *P < 0.05, **P < 0.01.

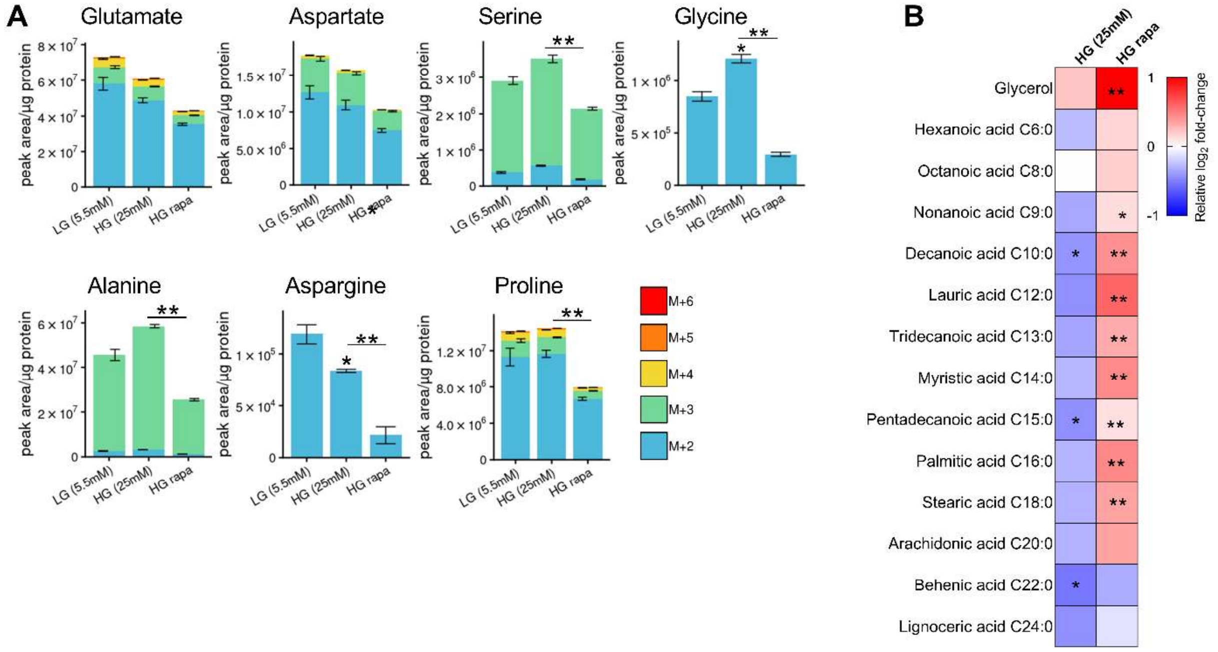

Supplemental Figure 5. (**A**) Relative abundance of ^{13}C-labeled glycogenic amino acids in KPTCs treated with LG HG or HG with rapamycin (10nM). (**B**) Heatmap showing the relative levels of free fatty acids in KPTCs. Each square indicates the average log2 fold change of metabolites relative to LG-treated KPTCs. n = 5 biological replicates. Data were analyzed by 1-way ANOVA. *P < 0.05, **P < 0.01.

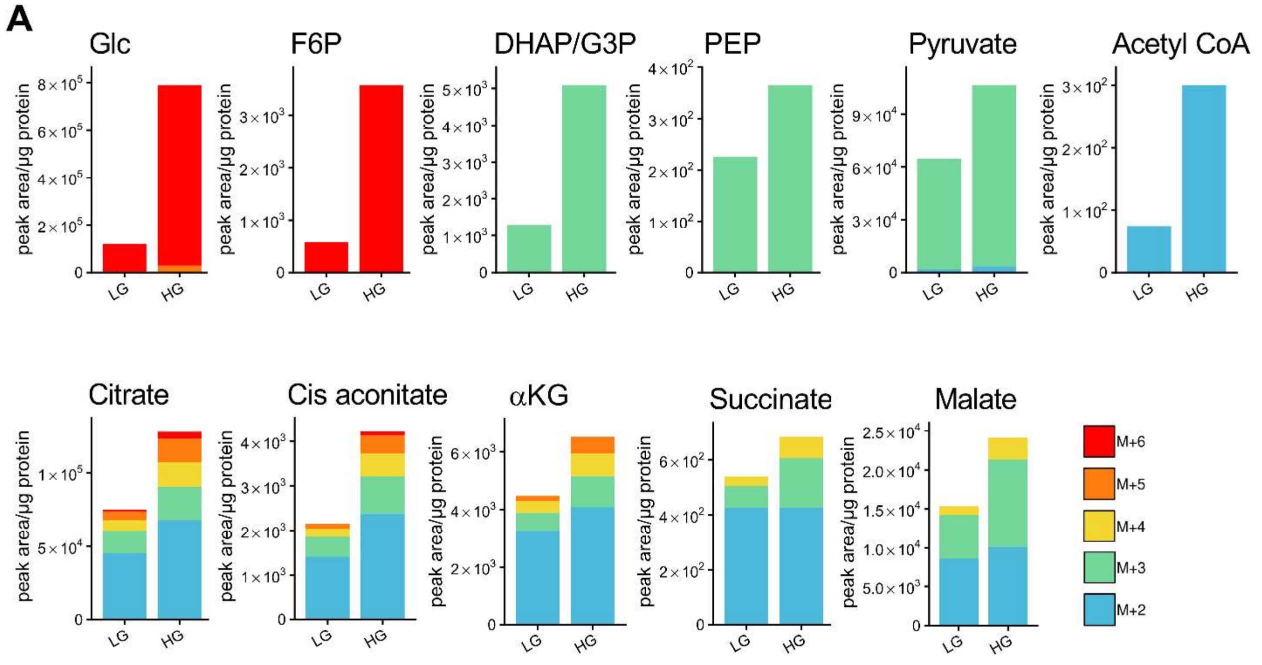

Supplemental Figure 6. $^{13}C_6$ glucose labeling of human islets exposed to LG or HG for 48h followed by metabolomics. The levels of labeled glucose, glycolysis, and TCA cycle metabolites are shown (n=2).

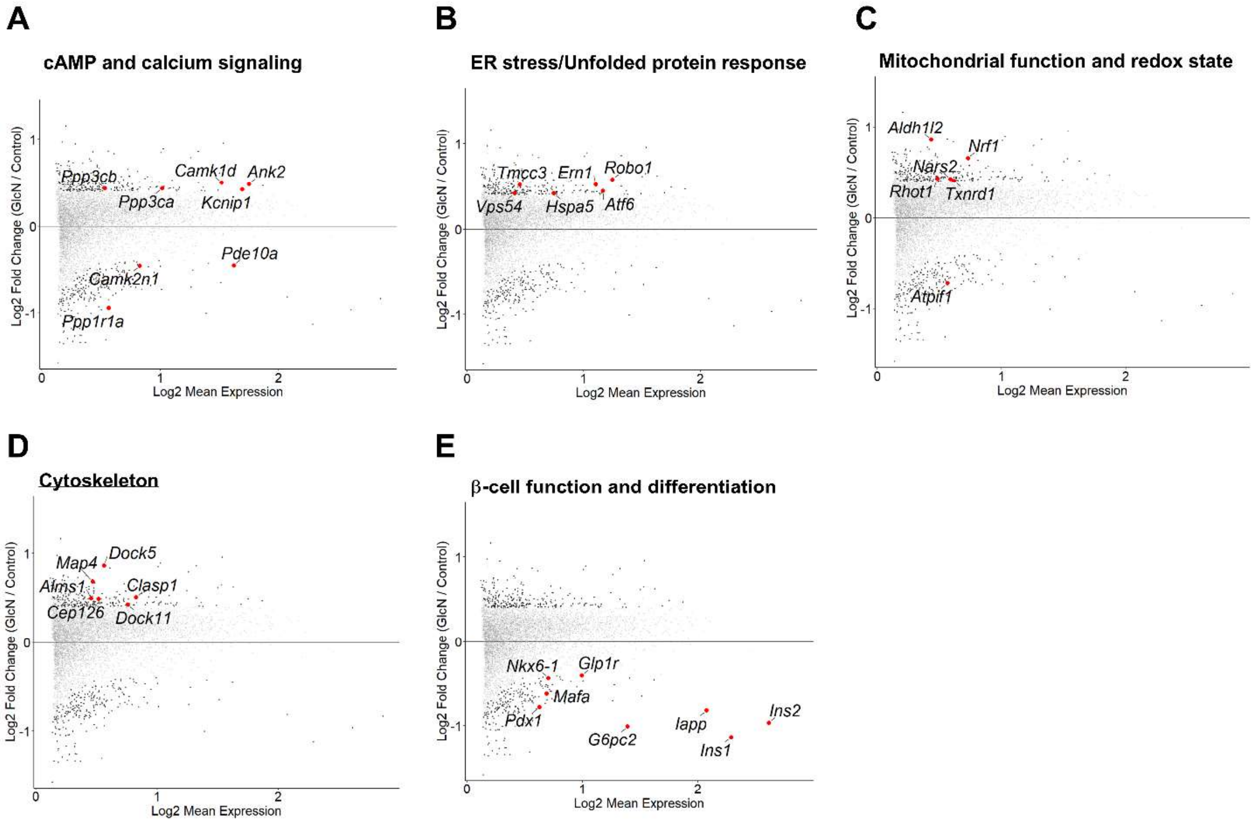

Supplemental Figure 7. MA plot of the glucosamine effect on the expression of genes regulating: (**A**) cAMP and calcium signaling, (**B**) ER stress/unfolded protein response, (**C**) mitochondria function and redox state, (**D**) cytoskeleton organization and (**E**) β-cell function and differentiation.

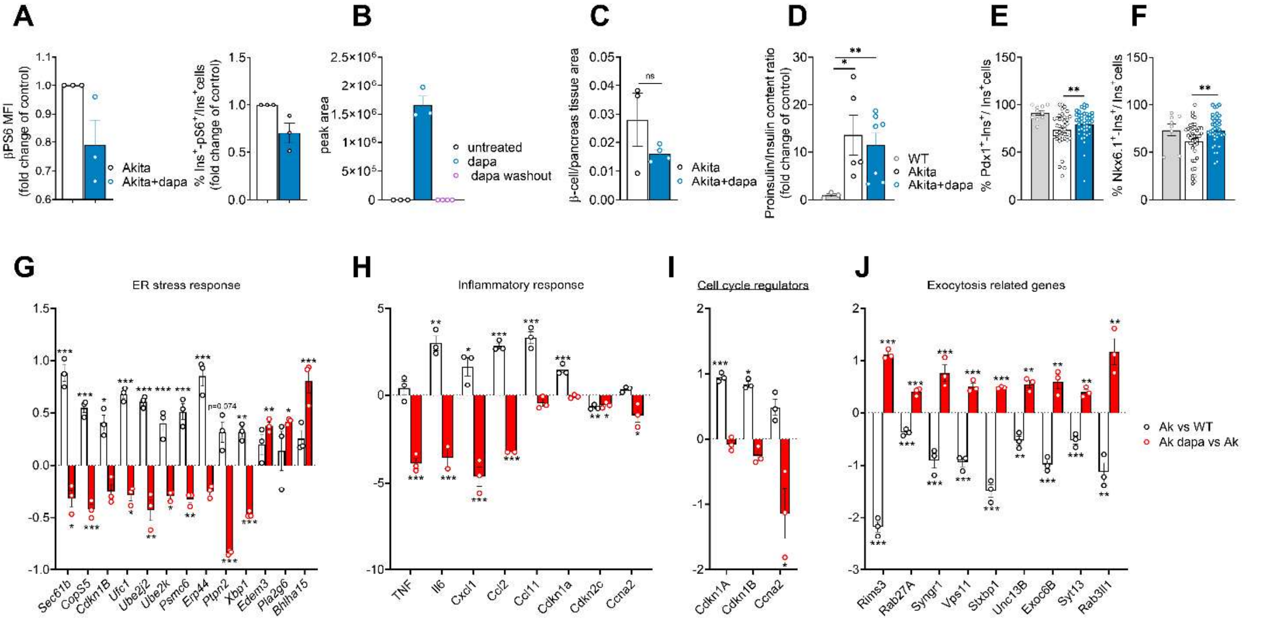

Supplemental Figure 8. 6-weeks-old Akita mice were treated with or without dapagliflozin (10 mg/kg/day in drinking water) for 2 weeks. (**A**) mTORC1 activity in β-cells was assessed by immunostaining of dispersed islets for $pS6^+$ and insulin followed by flow cytometry analysis. The percentage of $pS6^+$ β-cells and the mean fluorescence intensity (MFI) are shown (n=3) (**B**) Dapagliflozin was removed from drinking water for 48 h, followed by LCMS analysis for dapagliflozin to confirm complete drug washout (n=3) (**C**) Consecutive pancreatic sections were stained for insulin and β-cell area normalized to pancreatic area was quantified (n=3-4) (**D**) Pancreatic proinsulin/insulin content quantified by ELISA in Akita mice treated with or without dapagliflozin compared to WT controls (n=3-7 mice per group). (**E-F**) Pancreatic sections were stained for insulin and PDX-1 and NKX6.1(565-2615 β-cells were counted. (**G-J**) Expression of genes related to ER stress response (**G**), inflammatory response (**H**), cell cycle regulation (**I**) and exocytosis (**J**) in islets from 6-week-old WT mice, Akita mice, and Akita mice treated with dapagliflozin for 2 weeks. Results are presented as log2-fold change, comparing Akita vs. Data represent the mean ± SEM. Data were analyzed by 1-way ANOVA. *P < 0.05, **P < 0.01.
