# Supplementary material for: Glucosamine links hyperglycemia to mTORC1 activation and glucose toxicity in diabetes
Source: JCI Insight. 2026 May 22;11(10):e197331. doi: 10.1172/jci.insight.197331 (PMC13232723; doi:10.1172/jci.insight.197331)

Fig 3C

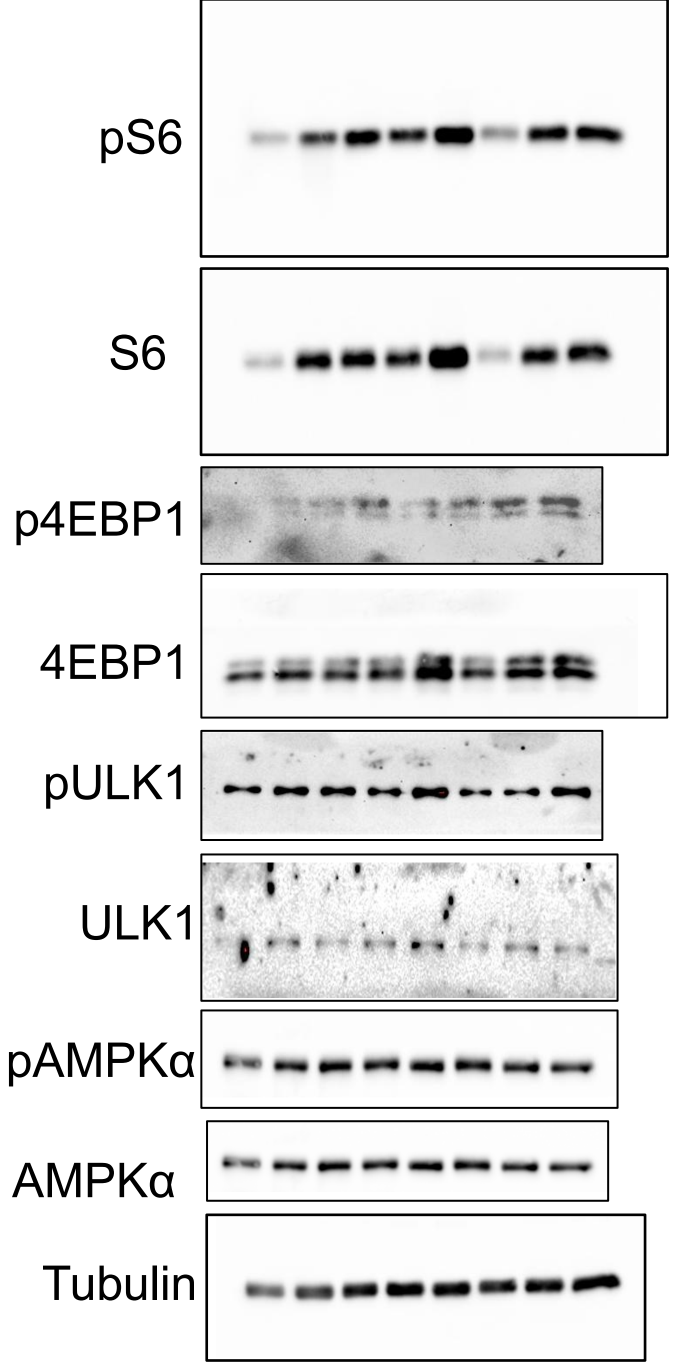

Fig 3D

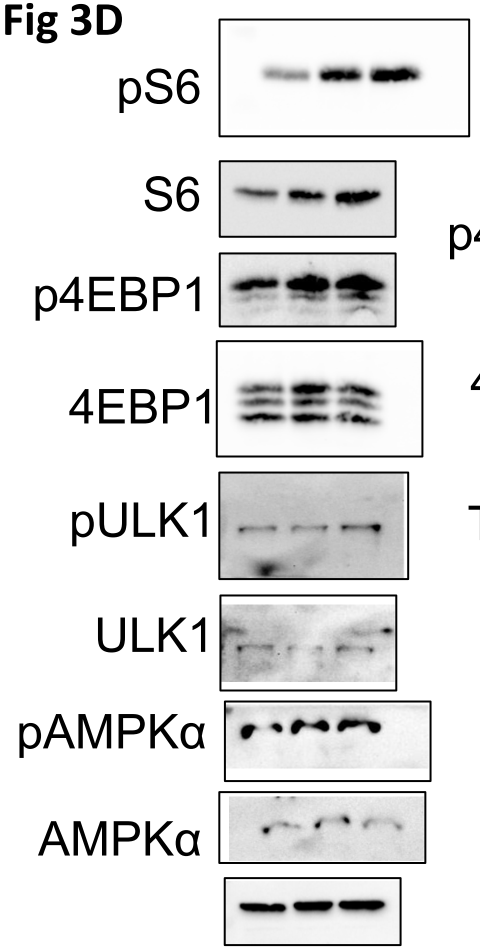

Fig 3E

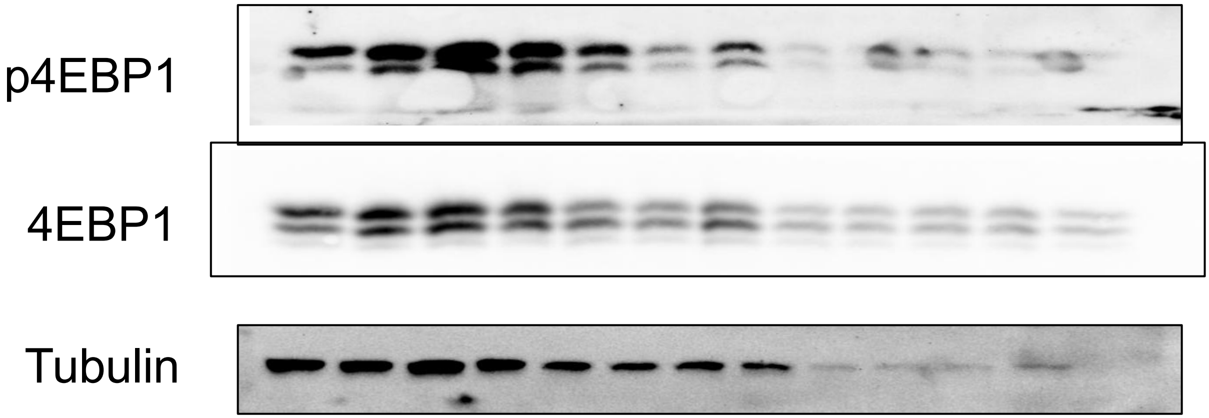

Fig 3G

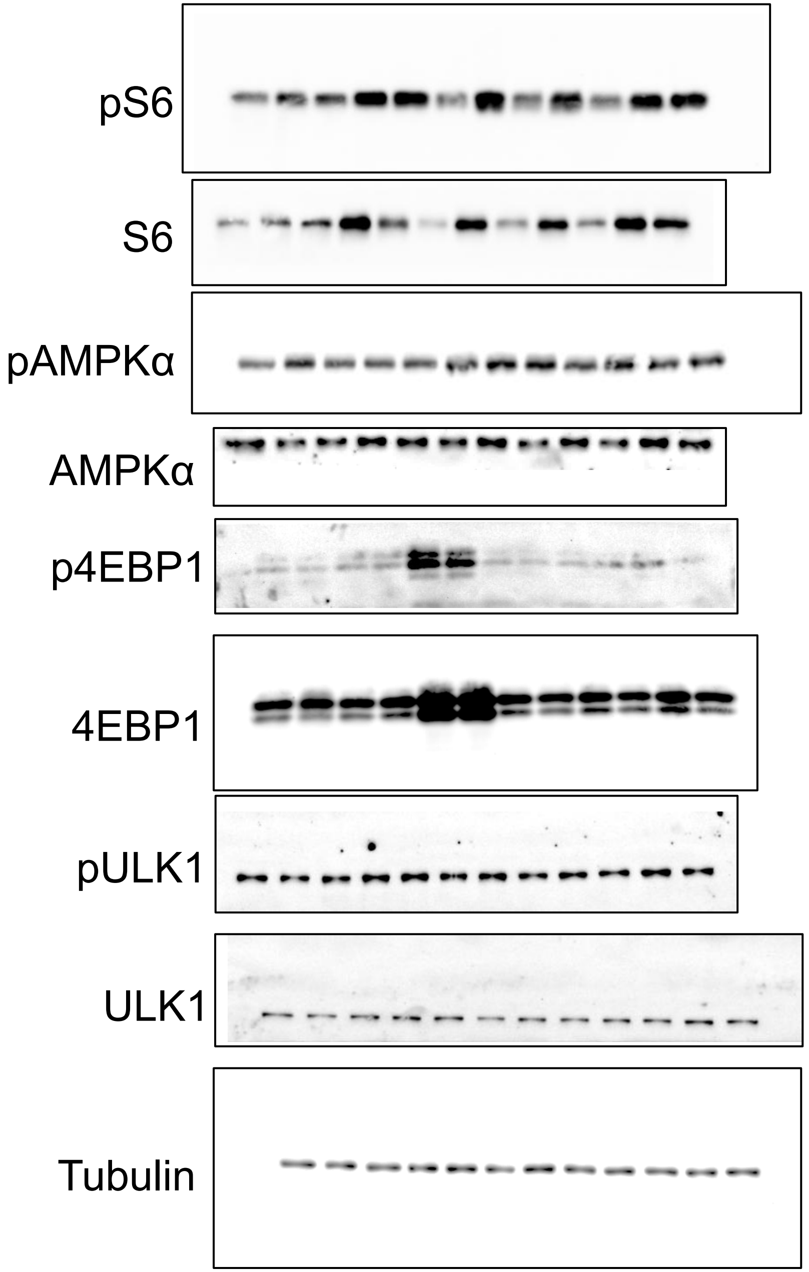

Supplemental Fig S2 A

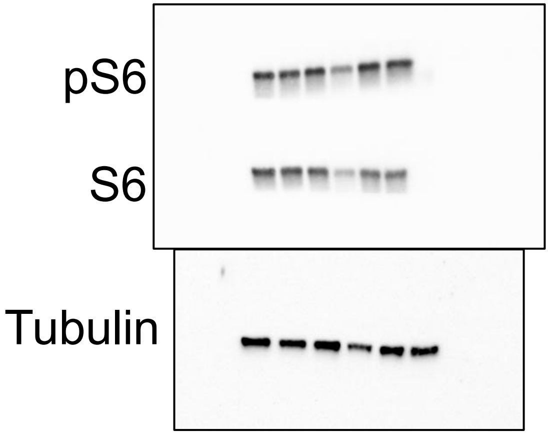

Supplemental Fig S2 B

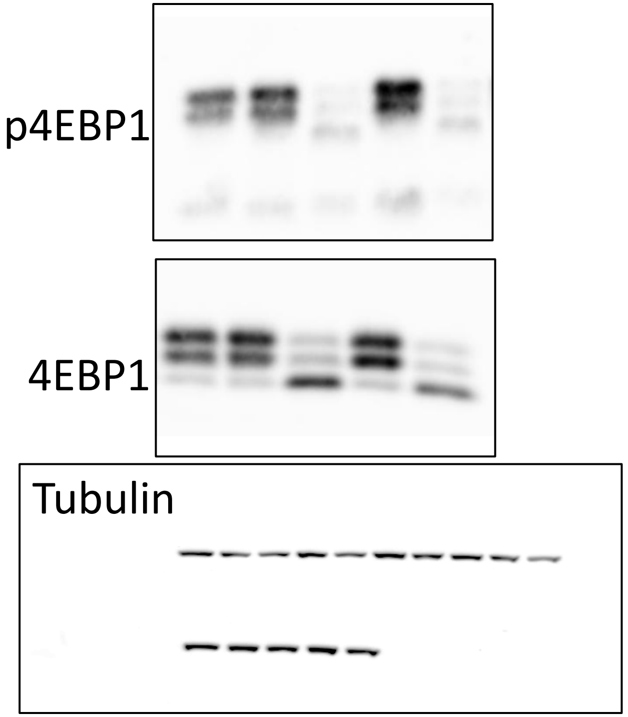

Fig 4A

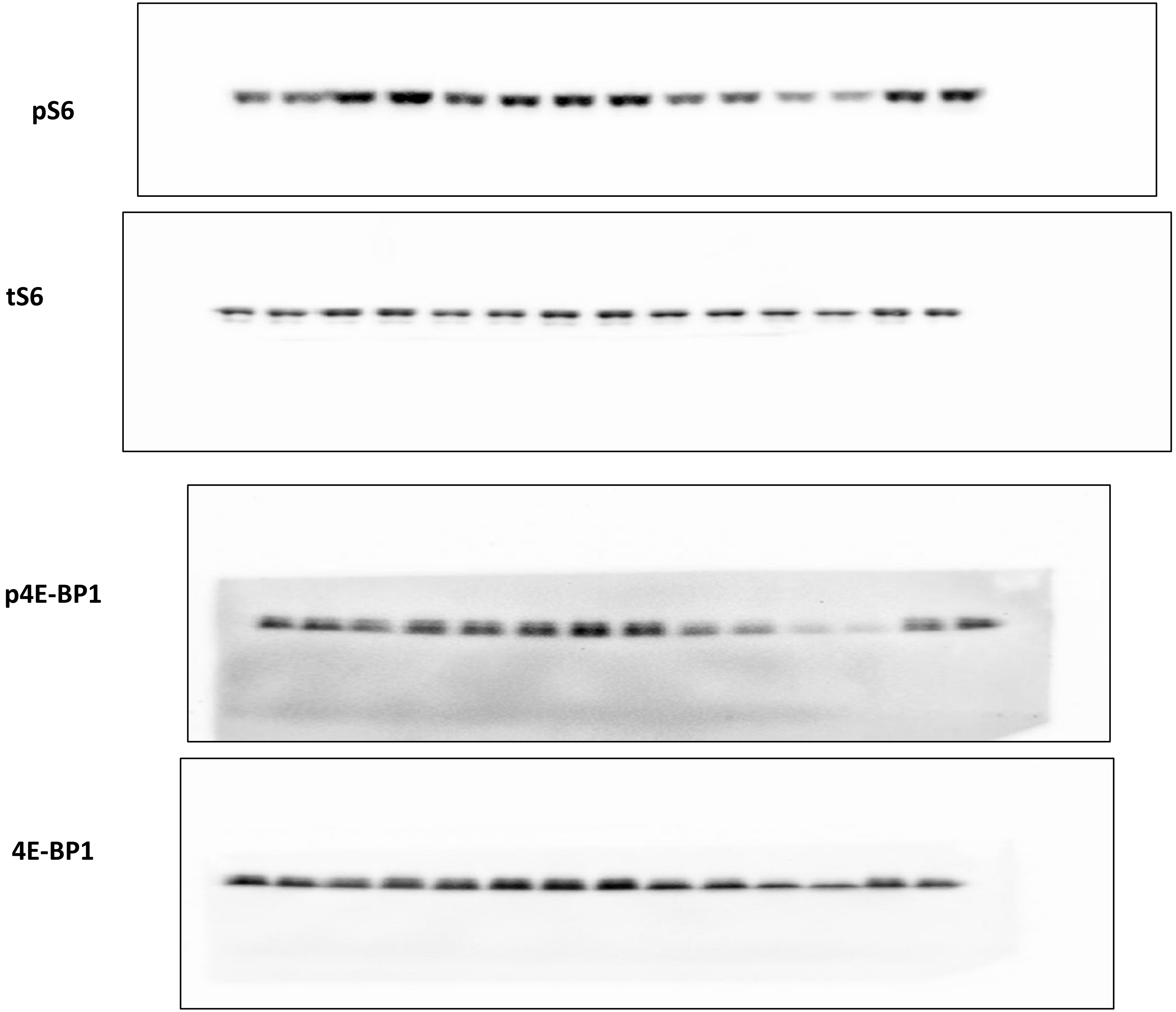

Fig 4A

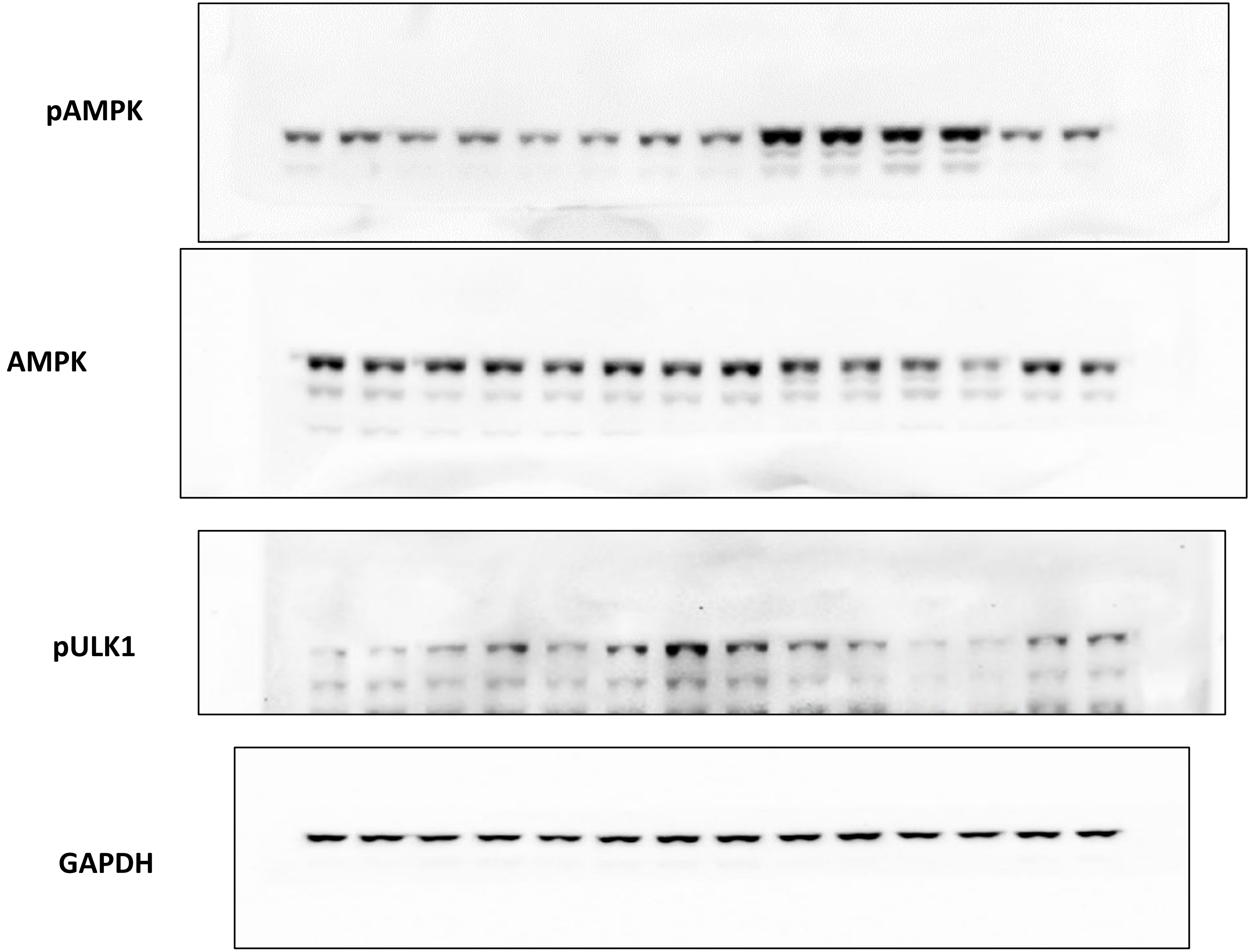

Fig 4B

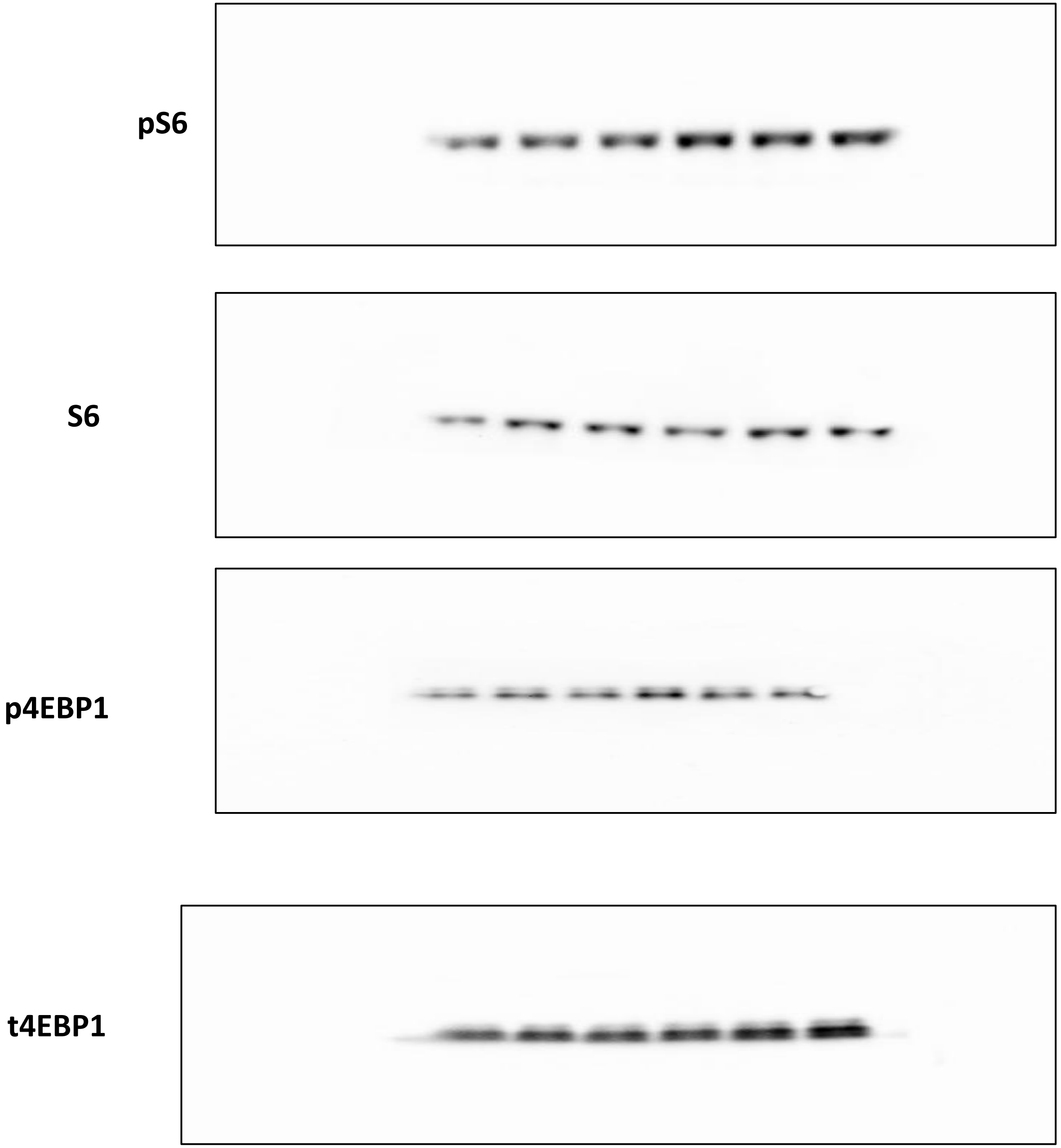

Fig 4B

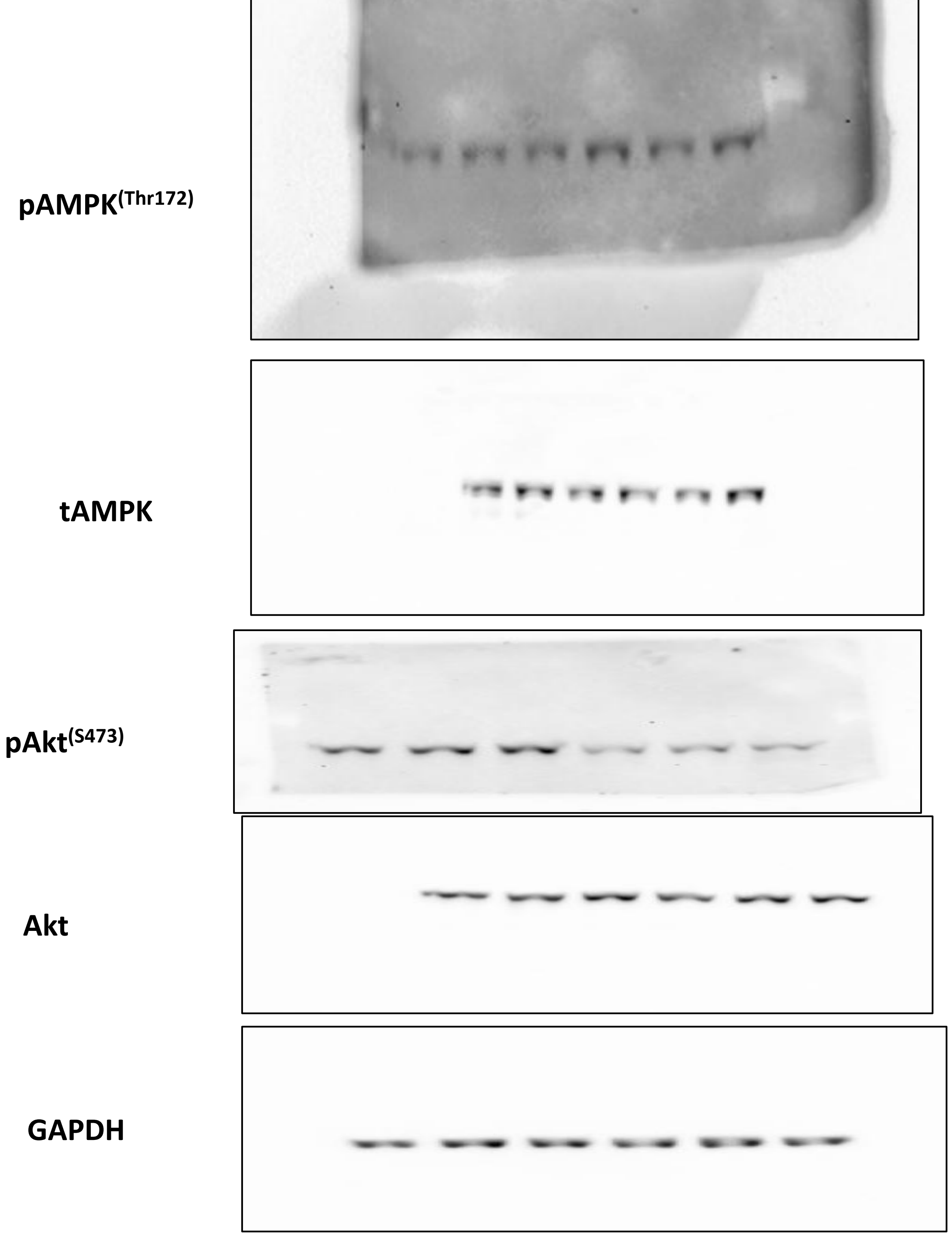

Supplement: Unedited blot and gel images [file jciinsight-11-197331-s101.pdf]
